# SlH3 and SlH4 promote multicellular trichome formation and elongation by upregulating *Woolly* in tomato

**DOI:** 10.1093/hr/uhaf008

**Published:** 2025-01-24

**Authors:** Seong-Min Kim, Da-Min Choi, Jae-In Chun, Seong-Yeop Kim, Seong-Hyeon Kim, Jeong-Il Kim, Ji-in Jang, Keunhwa Kim, Soon Ju Park, Jang-Kyun Seo, Choonkyun Jung, Jin-Ho Kang

**Affiliations:** Department of Agriculture, Forestry and Bioresources, College of Agriculture and Life Sciences, Seoul National University, Seoul 08826, Republic of Korea; Crop Biotechnology Institute, Institutes of Green-bio Science and Technology, Seoul National University, Pyeongchang 25354, Republic of Korea; Department of Integrative Food, Bioscience and Biotechnology, Chonnam National University, Gwangju 61186, Republic of Korea; Department of Agriculture, Forestry and Bioresources, College of Agriculture and Life Sciences, Seoul National University, Seoul 08826, Republic of Korea; Crop Biotechnology Institute, Institutes of Green-bio Science and Technology, Seoul National University, Pyeongchang 25354, Republic of Korea; Department of International Agricultural Technology, Seoul National University, Pyeongchang 25354, Republic of Korea; Integrated Major in Global Smart Farm, College of Agriculture and Life Sciences, Seoul National University, Seoul 08826, Republic of Korea; Department of Integrative Food, Bioscience and Biotechnology, Chonnam National University, Gwangju 61186, Republic of Korea; Department of Integrative Food, Bioscience and Biotechnology, Chonnam National University, Gwangju 61186, Republic of Korea; Plant Molecular Biology and Biotechnology Research Center (PMBBRC) and Division of Biological Sciences, Gyeongsang National University, Jinju 52828, Republic of Korea; Plant Molecular Biology and Biotechnology Research Center (PMBBRC) and Division of Biological Sciences, Gyeongsang National University, Jinju 52828, Republic of Korea; Plant Molecular Biology and Biotechnology Research Center (PMBBRC) and Division of Biological Sciences, Gyeongsang National University, Jinju 52828, Republic of Korea; Crop Biotechnology Institute, Institutes of Green-bio Science and Technology, Seoul National University, Pyeongchang 25354, Republic of Korea; Department of International Agricultural Technology, Seoul National University, Pyeongchang 25354, Republic of Korea; Department of Agriculture, Forestry and Bioresources, College of Agriculture and Life Sciences, Seoul National University, Seoul 08826, Republic of Korea; Crop Biotechnology Institute, Institutes of Green-bio Science and Technology, Seoul National University, Pyeongchang 25354, Republic of Korea; Department of International Agricultural Technology, Seoul National University, Pyeongchang 25354, Republic of Korea; Integrated Major in Global Smart Farm, College of Agriculture and Life Sciences, Seoul National University, Seoul 08826, Republic of Korea; Department of Agriculture, Forestry and Bioresources, College of Agriculture and Life Sciences, Seoul National University, Seoul 08826, Republic of Korea; Crop Biotechnology Institute, Institutes of Green-bio Science and Technology, Seoul National University, Pyeongchang 25354, Republic of Korea; Department of International Agricultural Technology, Seoul National University, Pyeongchang 25354, Republic of Korea; Integrated Major in Global Smart Farm, College of Agriculture and Life Sciences, Seoul National University, Seoul 08826, Republic of Korea

## Abstract

Trichomes are tiny outgrowths on the plant epidermis that serve defensive purposes against various stresses. While the regulatory mechanisms underlying unicellular trichome development are well understood, those governing multicellular trichome formation remain largely unexplored. In this study, we reveal a new regulatory pathway involving the *Hair3* (*H3*) and *H4* genes, which encode C2H2 zinc finger proteins that participate in multicellular trichome development in tomato (*Solanum lycopersicum*). Using CRISPR-Cas9 to generate single- and double-knockout lines, we found that *h3* and *h4* single-mutant plants did not show altered trichome characteristics compared to wild-type plants. However, *h3/h4* double-knockout plants displayed decreased densities of Types I, VI, and VII trichomes, increased densities of Types III and V trichomes, and reduced leaf and stem lengths of Type I trichomes, revealing that *H3* and *H4* redundantly regulate trichome development. Notably, protein interaction assays demonstrated that H3 and H4 formed both homo- and heterodimers, supporting their cooperative role. Transcriptome and gene expression analyses identified *H3* and *H4* as key regulators of several genes involved in trichome development, including *Woolly* (*Wo*) and its downstream targets, such as *Wox3b*, *MX1*, *H,* and *HD8*. Protein-promoter assays showed that H3 and H4 did not directly bind to the *Wo* promoter but rather interacted with Wo, thereby enhancing the expression of *Wo* and *Wox3b*. These findings establish *H3* and *H4* as key regulators of trichome development and provide novel insights into the mechanisms controlling multicellular trichome development in tomato plants.

## Introduction

Plant trichomes are surface outgrowths of epidermal cells that exhibit remarkable variability in size, shape, and distribution across plants. They defend against various biotic threats, including attacks from insects or pathogens [1–3], as well as abiotic stresses, such as high temperatures or excessive UV-B radiation [4, 5]. Trichomes are categorized as unicellular or multicellular based on the cell number or as glandular or nonglandular based on the presence of glands [6].

Arabidopsis (*Arabidopsis thaliana*) is an important model plant that has a unicellular nonglandular trichome that has been extensively studied in terms of the genetic and regulatory mechanisms governing its development. Notably, the MYB-bHLH-WDR (MBW) protein complex, composed of the R2R3-type MYB, basic helix–loop–helix (bHLH), and WD40 repeat proteins, initiates trichome development by regulating the expression of *GLABRA2* (*GL2*), which encodes the homeodomain leucine zipper IV (HD-ZIP IV) protein [7, 8]. In contrast, single-repeat R3-type MYB proteins, including Caprice (CPC) and Tryptichon (TRY), negatively regulate trichome development by competing with R2R3-type MYB proteins in their binding with bHLH proteins [9, 10]. Genes encoding the subunits of the MBW complex are controlled by several C2H2 *zinc finger protein* (*ZFP*) genes, such as *GLABROUS INFLORESCENCE STEMS* (*GIS*), *GIS2*, and *ZFP8* [11, 12]. These *ZFP* genes are regulated by other C2H2 *ZFP* genes, such as *GIS3*, *ZFP5*, and *ZFP6* [13–15], highlighting the critical role of C2H2 *ZFP* genes in *Arabidopsis* trichome development. In cotton, the development of single-cell fibers follows a mechanism similar to that in *Arabidopsis*. For example, the genes encoding MYB, bHLH, and WD40 initiate the development of cotton fibers and are homologous to those in *Arabidopsis* [16]. However, little is known about how these genes are regulated or whether *ZFPs* participate in their regulation.

Research on multicellular trichomes, particularly those with glandular characteristics, has predominantly focused on morphological classification, pest defense-related metabolite profiling, and pharmaceutical utilization across various plant species [17, 18]. Although the mechanisms underlying the development of multicellular trichomes have recently gained attention, the process remains largely unclear. Tomatoes, an important model crop plant, have seven types of multicellular trichomes that are classified based on the presence of gland heads: Types I, IV, VI, and VII are glandular, while Types II, III, and V are nonglandular [6]. Several transcription factor-encoding genes are believed to participate in the development of multicellular trichomes in tomato. *Woolly* (*Wo*), a member of the HD-ZIP IV family, was first identified as a key regulator of glandular trichomes [19]. *HOMEODOMAIN PROTEIN8* (*HD8*) is another HD-ZIP IV member that regulates trichome elongation [20, 21]. Genes such as *Hair (H)* and *H2* (*H2*, *SlZFP8-like*, *SH*, and *HL* denote identical genes; henceforth, referred to as *H2*), which encode C2H2 ZFPs, play tissue-specific regulatory roles in trichome development [22–26]. *Wo*-regulated genes, such as *WUSCHEL-related homeobox 3b* (*Wox3b*), *MIXTA-like 1* (*MX1*), and *Leafless* (*LFS*), determine the fate of distinct trichome types [27]. Lanata (Ln) and Wo regulate the expression of *CycB2* and *CycB3*, which negatively regulate trichome development [28, 29]. Interestingly, unlike in Arabidopsis, *THM1* and *MYB52*, which encode R2R3-type MYB proteins, negatively regulate trichome initiation in tomato [3]. Although several pivotal genes have been identified in tomatoes, the regulatory mechanisms underlying multicellular trichome development and function remain elusive.

**Figure 1:**
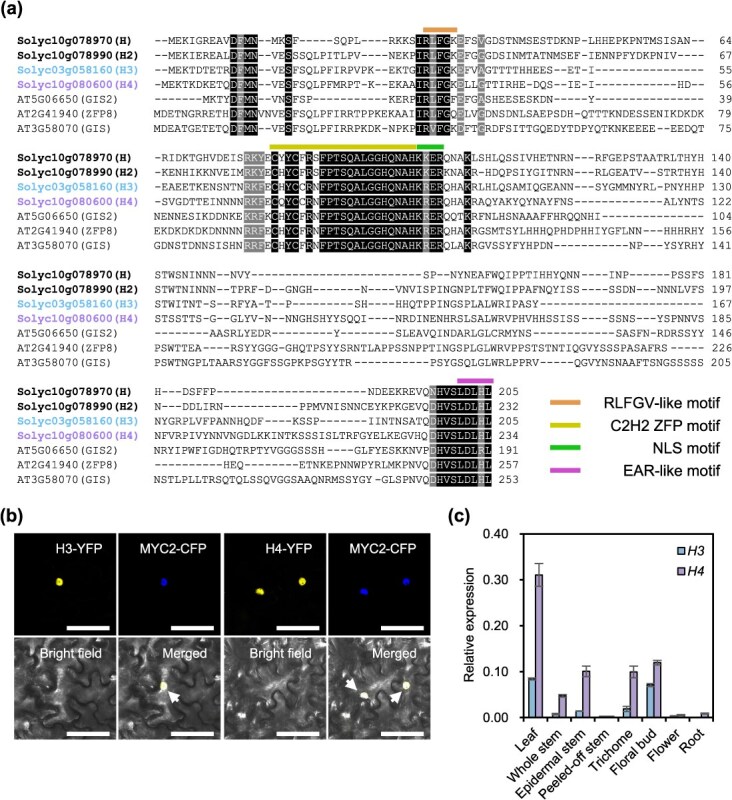
H3 and H4 are ZFPs. (a) Amino acid sequence alignment of H3, H4, and closely related homologs in tomato and *Arabidopsis*. Proteins belonging to the same groups as H3 and H4 are shown and compared in Fig. S1. Black and gray shaded areas indicate regions with identical and highly conserved amino acids (>90% similarity). Colored lines highlight conserved motifs. (b) Subcellular localization of H3 and H4. Agroinfiltration was used to coinfiltrate MYC2-CFP with either H3-YFP or H4-YFP constructs in tobacco leaves. Arrows indicate overlapping regions between MYC2-CFP and H3-YFP or H4-YFP. Scale bar: 20 μm. (c) qRT-PCR analysis of *H3* and *H4* expressions in various tissues of WT plants. Data are presented as the mean (± standard error [SE]) of three biological replicates.

In this study, we aimed to identify and characterize the *H3* and *H4* genes in tomato plants. The study findings reveal the crucial roles of *H3* and *H4* in multicellular trichome development in tomatoes and provide new insights into the regulatory mechanisms governing trichome development.

## Results

### *H3* and *H4* encoded C2H2 zinc finger proteins

ZFPs are crucial for plant development, including trichome formation. Previous studies have demonstrated the involvement of *H* and its closely related homolog *H2* in initiating Type I trichome development in tomatoes [22, 23]. Phylogenetic analysis was performed to identify the potential *ZFP* genes involved in trichome development. The analysis utilized tomato and Arabidopsis ZFP, which exhibited high similarity to H and H2 (Fig. S1). The two tomato proteins Solyc03g058160 and Solyc10g080600 (hereafter termed H3 and H4) were clustered within the same group as H and H2 (Fig. S1). Amino acid sequence analysis revealed the conservation of key motifs, including C2H2 ZFP, nuclear localization signal (NLS), RLFGV-like, and EAR-like motifs, across this clade, including H3 and H4 (Fig. 1a). Subcellular localization studies using yellow fluorescent protein (YFP)-fused H3 and H4 in tobacco leaves confirmed their nuclear localization (Fig. 1b). Quantitative reverse-transcription polymerase chain reaction (qRT-PCR) was then performed to examine the expression patterns of *H3* and *H4* in wild-type (WT) plants across various tissues. *H3* and *H4* were predominantly expressed in the leaves, floral buds, and stems. Within stems, expression levels were significantly higher in epidermal stems than in stems where the epidermis had been peeled off. In isolated trichomes, *H3* and *H4* expression was comparable to that in epidermal stems. Overall, *H4* displayed higher expression levels than *H3* (Fig. 1c).

### *H3* and *H4* regulated the formation and elongation of trichomes in a redundant manner

To investigate the involvement of *H3* and *H4* in trichome initiation, *h3* and *h4* single-knockout (sko) plants were generated using the CRISPR-Cas9 system and *Agrobacterium*-mediated tomato transformation [30, 31]. Among the 14 *h3* sko and 12 *h4* sko T_0_ plants, two representative T_2_ lines exhibiting homozygous mutations resulting in premature stop codons and lacking the *Cas9* gene were selected for each genotype (*h3* sko: *h3*-sko-10 and *h3*-sko-14; *h4* sko: *h4*-sko-1 and *h4*-sko-12) (Fig. S2). Sequence analysis confirmed the absence of mutations in the closely related nontarget genes (*H*, *H2*, *H3*, and *H4*) in the selected sko lines. The selected lines were then subjected to trichome phenotyping. Microscopy examination revealed no observable changes in trichome morphology, number, or length on the leaves and stems of *h3* sko and *h4* sko plants compared to WT plants (Figs S3 and S4).

**Figure 2:**
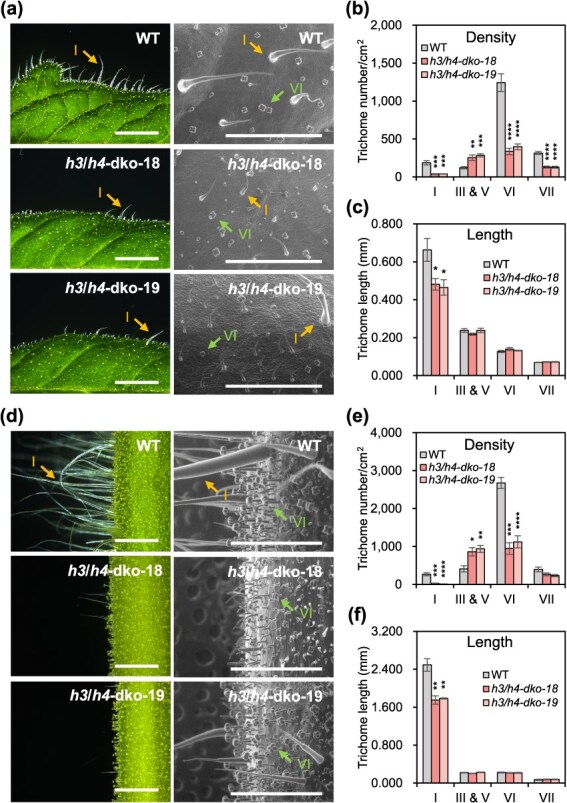
Trichome phenotypes of *h3/h4* dko plants. (a) Dissection microscopy (DM) and scanning electron microscopy (SEM) images of the leaves of WT and *h3*/*h4* dko plants. Scale bars: 2 mm (DM) and 1 mm (SEM). (b, c) Trichome density (b) and length (c) on the leaves of WT and *h3*/*h4* dko plants. (d) DM and SEM images of stems in WT and *h3/h4* dko plants. Scale bars: 2 mm (DM) and 1 mm (SEM). (e, f) Trichome density (e) and length (f) on the stems of WT and *h3/h4* dko plants. All images were taken from 6-week-old plants. Data are presented as the mean (±SE) of six biological replicates. Asterisks indicate significant differences between WT and *h3/h4* dko plants (unpaired *t*-test: ^*^P < 0.05, ^**^P < 0.01, ^***^P < 0.001, ^****^P < 0.0001).

Given the absence of trichome-related phenotypes in *h3* sko and *h4* sko plants, we hypothesized that *H3* and *H4* function redundantly. To validate this hypothesis, *h3*/*h4* double (d)-ko plants were generated using the multiple ko CRISPR-Cas9 system and *Agrobacterium*-mediated tomato transformation [30, 32]. Among the 19 *h3*/*h4* dko T_0_ plants, two representative T_2_ lines (*h3*/*h4*-dko-18 and *h3*/*h4*-dko-19) were selected, both of which exhibited homozygous mutations, leading to premature stop codons and a lack of *Cas9* (Fig. S5). Similar to the sko plants, sequence analysis revealed no mutations in the *H* or *H2* genes in the selected *h3*/*h4* dko lines. In contrast to the *h3* sko and *h4* sko plants, *h3*/*h4* dko plants displayed trichome defects. The leaves and stems of *h3/h4* dko plants exhibited reduced numbers of Type I, VI, and VII trichomes but increased numbers of Type III and V trichomes compared to WT plants (Fig. 2a, b, d, and e). Additionally, while the lengths of most trichome types remained unchanged, the lengths of Type I trichomes were significantly reduced in *h3*/*h4* dko plants compared with those in WT plants (Fig. 2c and f). Further analysis indicated that this reduction in Type I trichome length resulted from a decrease in stalk cell number because most individual cell lengths remained consistent with those in WT plants (Fig. S6). Trichome density and morphology in the sepals remained unchanged between the WT and sko and dko plants. However, the trichome density and length in the hypocotyl were lower in *h3*/*h4* dko plants than those in WT and sko plants (Fig. S7). These results indicated that *H3* and *H4* contribute complementarily to trichome development across various tomato tissues.

### *H3* and *H4* were implicated in plant, leaf, and epidermal cell development

Plant growth rates were similar between *h3* sko and WT plants, whereas *h4* sko plants were smaller (Fig. S8a and b). In contrast, *h3/h4* dko plants exhibited reduced plant size, smaller leaves, and a more rounded leaf shape than WT plants (Fig. S8c–f). We investigated the cause of altered leaf size and observed a decrease in epidermal cell density per unit area in *h3/h4* dko plants relative to WT and sko plants (Fig. S9a). Moreover, unlike the convex and smooth epidermal cells observed in the WT and sko plants, the epidermal cells in the *h3/h4* dko plants appeared flatter and more wrinkled (Fig. S9b). These results indicated that *H3* and *H4* are involved in regulating plant growth and epidermal cell development.

### H3 and H4 formed homo- and heterodimers

**Figure 3:**
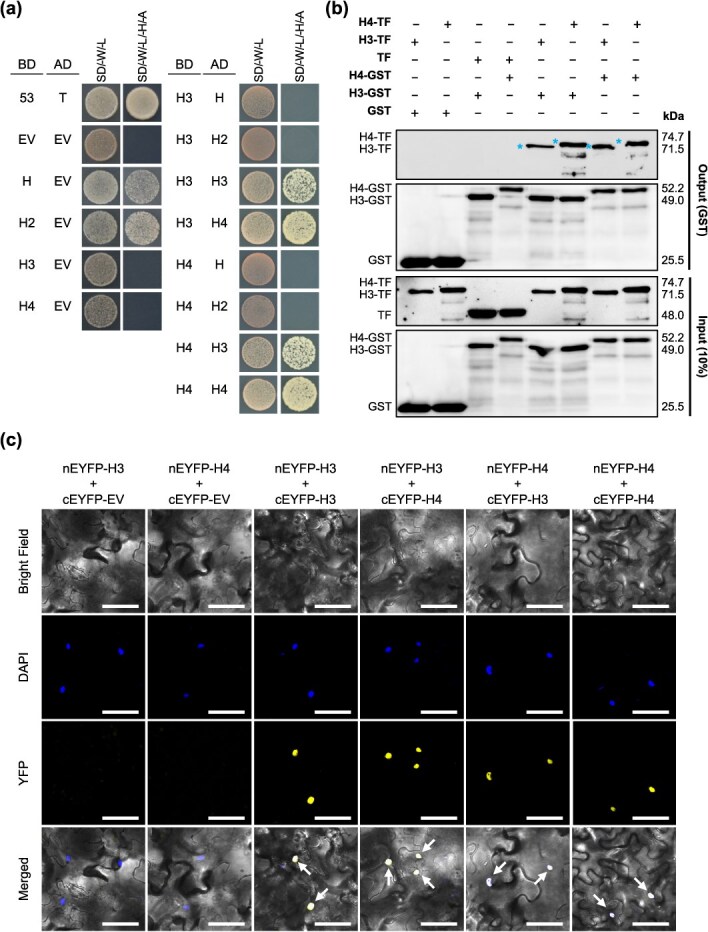
H3 and H4 homodimer and heterodimer formation. (a) Y2H assays assessing PPIs between H3 and H4. Full-length H, H2, H3, and H4 were fused downstream of the GAL4 DNA-binding domain (BD) in pGBKT7 (H-BD, H2-BD, H3-BD, and H4-BD, respectively) and cotransformed with a pGADT7 empty vector (EV) containing the GAL4 DNA activation domain (AD; EV-AD) or full-length H, H2, H3, or H4 were fused with pGADT7 (H-AD, H2-AD, H3-AD, or H4-AD) in *S. cerevisiae* strain AH109. Yeast cells containing 53-BD/T-AD or EV-BD/EV-AD vectors were used as positive and negative controls, respectively. Growth was observed in double (SD/-W/-L) and quadruple (SD/-W/-L/-H/-A) dropout media. (b) *In vitro* PPI analysis using GST pull-down assay. Purified His/TF-tagged H3 or H4 protein (H3-TF or H4-TF) or TF protein alone were incubated with GST/streptavidin (Strep)-tagged H3 or H4 protein (H3-GST or H4-GST) or GST protein. Combinations of GST or TF proteins served as negative controls. Proteins bound to glutathione beads were analyzed via western blotting using anti-TF or anti-GST antibodies. Blue asterisks denote the expected sizes of H3-TF or H4-TF. (c) *In planta* PPI analysis using BiFC assay. nEYFP-(H3 or H4) and cEYFP-(H3 or H4) were coinfiltrated in tobacco leaves via agroinfiltration. nEYFP-(H3 or H4) with empty cEYFP (cEYFP-EV) constructs served as negative controls. Arrows indicate the overlapping signals of 4′,6-diamidino-2-phenylindole (DAPI) and YFP. Scale bars: 60 μm.

ZFPs can function through protein interactions [33]. Considering the redundant functions of *H3* and *H4*, they likely interact with each other. Thus, we conducted yeast two-hybrid (Y2H) experiments to investigate the dimerization potential of H3 and H4 proteins. Compared with the full-length H and H2 proteins, which exhibited autoactivation activity, the full-length H3 and H4 proteins did not show inherent autoactivation (Fig. 3a). Interaction assays revealed that H3 and H4 formed both homodimers and heterodimers but did not interact with H or H2 (Fig. 3a). Pull-down assays were performed to validate these interactions *in vitro*, and they demonstrated that H3-glutathione *S-*transferase (GST) precipitated H3-trigger factor (TF) and H4-TF. Similarly, H4-GST precipitated H3-TF and H4-TF (Fig. 3b). Bimolecular fluorescence complementation (BiFC) assays were used to examine dimerization *in planta*. The coexpression of nEYFP-H3 with cEYFP-H3 or cEYFP-H4 and nEYFP-H4 with cEYFP-H3 or cEYFP-H4 resulted in detectable YFP activity, thus confirming the homo- and heterodimerization of H3 and H4 (Fig. 3c) and suggesting cooperative interactions in their regulatory functions.

### *H3* and *H4* modulated the expression of several key genes associated with trichome development

**Figure 4:**
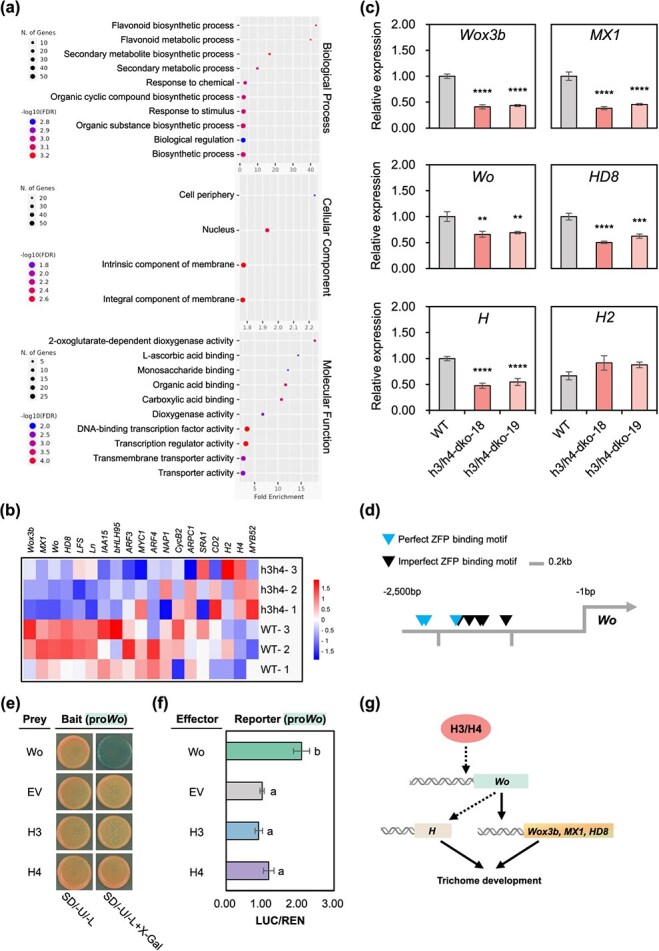
Transcriptomic analysis of *h3/h4* dko plants. (a) GO enrichment analysis of DEGs between *h3/h4* dko and WT leaves (|log_2_FoldChange| ≥ 1, FDR-adjusted *P* < 0.01). The left *y*-axis indicates each GO term, while the *x*-axis represents the fold enrichment value. (b) Heat map displaying the levels of representative trichome-related genes in *h3/h4* dko and WT leaves. The scale bar represents the log_2_FoldChange value of each gene. (c) qRT-PCR analysis of trichome-related genes in *h3/h4* dko and WT leaves. Gene expression was normalized to that in WT plants. Data are presented as the mean (±SE) of three biological replicates. Each replicate comprises three leaves pooled together. ^**^P < 0.01, ^***^P < 0.001, ^****^P < 0.0001 (unpaired Student’s *t*-test). (d) Schematic diagram of the promoter region (2.5 kb) of *Wo*. Blue/black triangles indicate perfect/imperfect ZFP binding sites. Scale bar: 200 bp. (e) Analysis of H3 and H4 proteins binding to *Wo* promoters using Y1H assay. The *Wo* promoters were fused to the pLacZi bait vector (pLacZi-pro*Wo*). Full-length Wo, H3, and H4 were fused downstream of the GAL4 DNA AD in pGADT7. Constructs containing the pGADT7 EV were used as negative controls. Linearized bait vectors were integrated into the yeast genome, and prey vectors were transformed into the corresponding yeast strain, YM4271. The transformants were incubated on an SD/-Ura/-Leu dropout medium for 3 days and transferred onto an SD/-Ura/-Leu dropout medium containing X-gal. (f) Transactivation analysis of *Wo* promoters by H3 and H4 proteins using DLR assay. The *Wo* promoter was cloned upstream of LUC into the pGreen II-0800-LUC reporter vector (pGreen II-pro*Wo*). Full-length Wo, H3, and H4 were fused downstream of the 35S promoter in the pKCo effector vectors (*Wo*, *H3*, and *H4*, respectively). Both reporter and effector constructs were coinfiltrated in tobacco leaves via agroinfiltration. Constructs containing an empty pKCo vector (35S:EV) were used as negative controls. The DLR assay was performed 2 days postinfiltration. LUC activity was quantified using the value of LUC relative to that of each reporter vector and normalized to that of the negative control. Data are presented as the mean (±SE) of eight biological replicates. Different letters indicate statistically significant differences (*P* < 0.05, one-way analysis of variance [ANOVA] with Tukey’s *post hoc* test). (g) Schematic diagram showing representative trichome-related genes regulated by *H3* and *H4*. H3 and H4 indirectly regulate the expression of *Wo*. The Wo protein, in turn, directly regulates the expressions of *Wox3b*, *MX1*, and *HD8* ( [27]; Fig. S19; solid arrows) and indirectly regulates the expression of *H* ([29]; dotted arrows).

To understand how *H3* and *H4* mutations affect trichome development at the molecular level, RNA sequencing (RNA-Seq) was conducted using leaf tissues from *h3*/*h4* dko and WT plants. Among the 35 768 standard genes (ITAG4.0) assessed for transcriptomic analysis, 23 101 were detected, with 448 differentially expressed genes (DEGs) identified between the *h3*/*h4* dko and WT plants. Of the DEGs, 258 were upregulated and 190 were downregulated in *h3*/*h4* dko plants compared to those in WT plants (Table S1). Gene Ontology (GO) enrichment analysis revealed that *H3* and *H4* mutations influenced various biosynthetic processes and transcription-related activities, including DNA-binding transcription factor and transcription regulator activities (Fig. 4a). These findings suggest that the absence of functional *H3* and *H4* affect multiple biological and molecular processes, highlighting their intricate regulatory roles in trichome development.

We investigated the regulatory roles of *H3* and *H4* genes based on the levels of 19 key regulatory genes implicated in trichome development in tomato plants. Notably, *Wox3b*, *MX1*, *Wo*, and *HD8* exhibited significantly reduced expression in *h3/h4* dko plants compared to WT plants. The levels of other genes were either slightly different or comparable between *h3/h4* dko and WT plants. *H* gene expression was not detectable in the RNA-Seq data due to low read counts, while *H2* showed limited expression, making its expression pattern challenging to ascertain (Fig. 4b and Table S2). qRT-PCR confirmed these findings, showing a significant reduction in the levels of *Wox3b*, *MX1*, *Wo*, and *HD8* in *h3/h4* dko compared with those in WT plants (Fig. 4c). A comparison of gene expression patterns using specific primers revealed a significant reduction in *H* expression in *h3/h4* dko plants compared with that in WT plants, whereas *H2* expression remained unchanged (Fig. 4c). We investigated the regulatory effects of *H3* or *H4* single mutations on these genes and found that, except for *Wox3b* and *HD8*, which displayed reduced expression in *h4* sko plants, none of the genes were regulated by any mutation (Fig. S10).

Previous studies have indicated that Wo regulates the expression of *Wox3b* and *MX1* directly [27] and that of *H* indirectly [29]. Our results demonstrated a decrease in the expression of *Wo* in *h3/h4* dko plants (Fig. 4c). To explore whether *Wo* regulates the expression of *H3* and *H4* through a feedback loop, we generated *wo* sko plants using the CRISPR-Cas9 system. The *wo* sko lines displayed fewer trichomes across most types, including Type I, in both the leaves and stems than WT plants. While the lengths of most trichome types were unaffected, Type I trichomes were significantly shorter in *wo* sko plants than in WT plants (Figs S11 and S12). Further analysis showed that this reduction in Type I trichome length was due to a decrease in stalk cell number and reduction in the length of each cell (Fig. S13). The expressions of *H* and *H2* were lower in *wo* sko plants than that in WT plants. However, the levels of *H3* and *H4* remained similar between *wo* sko and WT plants (Fig. S14). These results indicate that *H3* and *H4* collectively regulate the expression of *Wo* and that *Wo* does not transcriptionally regulate *H3* and *H4*.

Notably, the promoter region of *Wo* (~2.5 kb) harbored multiple C2H2 ZFP-binding sites (A[AG/CT] CNAC) [14] (Fig. 4d). To investigate whether *Wo* is a direct target of H3 and H4, we performed yeast one-hybrid (Y1H) and dual-luciferase reporter (DLR) assays and found that Wo can self-activate its own expression (Fig. 4e and f), consistent with previous findings [27]. However, H3 and H4 showed no blue coloration in the X-gal-containing medium (Fig. 4e) or luciferase activity (Fig. 4f), suggesting that H3 and H4 did not directly regulate *Wo* expression. Several Arabidopsis ZFPs have been shown to control trichome development by regulating other *ZFP* genes [13]. Given the reduced expression of *H* in *h3/h4* dko plants (Fig. 4c), we investigated whether *H* regulates the expression of *H3* and *H4* through a feedback loop. Using the CRISPR-Cas9 system, we generated *h* sko plants. The *h* sko mutant lines exhibited a reduced number of Type I trichomes in both the leaves and stems but showed an increase in Type III and V trichomes on the stems compared to WT plants. Additionally, Type I trichomes on the leaves and stems were shorter in the *h* sko mutant than in the WT plants (Figs S15 and S16). Further analysis indicated that this reduction in Type I trichome length was due to a decrease in stalk cell number and reduction in length of each cell (Fig. S17). The levels of *H3* and *H4* were similar between *h* sko and WT plants (Fig. S18a), indicating that *H3* and *H4* act upstream of *H* and that *H* does not regulate *H3* and *H4* through a feedback mechanism. The Y1H and DLR assays did not show the direct regulation of the *H* gene by H3 and H4 proteins (Fig. S18b–d). Since *HD8* has been implicated in regulating trichome length in tomato [20, 21] and displayed reduced expression in *h3/h4* dko plants (Fig. 4c) and *wo* sko plants (Fig. S19a), we assessed whether *HD8* is a direct target of H3, H4, and Wo. The Y1H assay showed no color change for H3 and H4 on plates containing X-Gal or after colony-lift assay, indicating that these proteins do not directly interact with the *HD8* promoter. In contrast, Wo exhibited a blue color change after colony-lift assay, suggesting a direct interaction with the HD8 promoter. DLR assays further confirmed that Wo regulates *HD8* expression while H3 and H4 do not (Fig. S19b–d). These results imply that Wo directly regulates *HD8* while H3 and H4 regulate *HD8* indirectly. These findings suggest that *H3* and *H4* contribute to trichome development by regulating various trichome developmental genes, partially through the modulation of *Wo* expression, which acts as a master regulator of several genes (Fig. 4g).

### H3 and H4 interacted with Wo and enhanced the transcription of Wo-regulated genes

**Figure 5:**
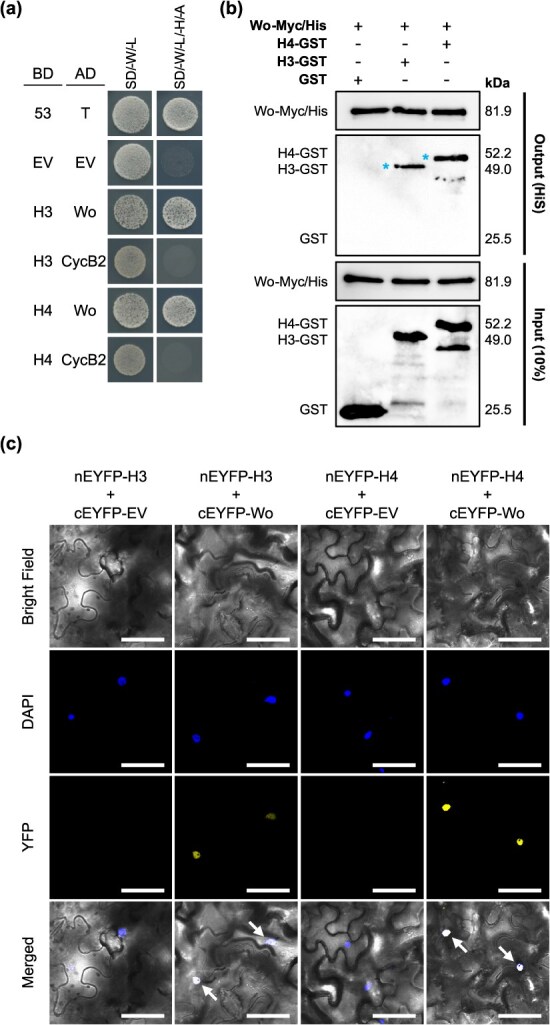
H3 and H4 interact with Wo. (a) Y2H analysis of PPIs. Yeast cells (AH109) were cotransformed with H3-BD or H4-BD along with full-length Wo or CycB2 fused to pGADT7 (Wo-AD or CycB2-AD). Positive controls included cells transformed with 53-BD/T-AD, while negative controls had EV-BD/EV-AD vectors. Growth was assessed in double (SD/-W/-L) and quadruple (SD/-W/-L/-H/-A) dropout media. (b) *In vitro* PPI analysis using pull-down assays. Myc/His-tagged Wo protein (Wo-Myc/His) was incubated with GST/Strep-tagged H3 or H4 (H3-GST or H4-GST) or GST protein. Negative controls included combinations of the GST proteins. Ni-NTA bead-bound proteins were analyzed via western blotting using anti-Myc and anti-GST antibodies. Blue asterisks indicate the expected sizes of H3-GST and H4-GST. (c) *In planta* PPI analysis using BiFC assay. Agroinfiltration of tobacco leaves was performed with nEYFP-(H3 or H4) and cEYFP-Wo. Negative controls included nEYFP-(H3 or H4) with empty cEYFP (cEYFP-EV). Arrows highlight the overlapping region between the DAPI and YFP signals. Scale bar: 60 μm.

As H3 and H4 do not directly regulate *Wo*, we investigated whether H3 and H4 interacted with Wo to influence trichome development. Y2H interaction assays indicated that both H3 and H4 interacted with Wo, but not with CycB2, a protein known to interact with Wo ( [19]; Fig. 5a). We performed pull-down assays to confirm these interactions and found that Wo-Myc/His precipitated both H3-GST and H4-GST (Fig. 5b). BiFC assays also demonstrated that the coexpression of nEYFP-H3 with cEYFP-Wo and nEYFP-H4 with cEYFP-Wo resulted in detectable YFP activity, confirming the direct interaction of H3 and H4 with Wo (Fig. 5c). Furthermore, pull-down assays involving Wo, H3, and H4 showed that all three proteins were coprecipitated (Fig. S20, last lane), suggesting the potential formation of a trimeric complex. These findings indicate that H3 and H4 might function cooperatively with Wo, thereby enhancing their regulatory roles in trichome development.

**Figure 6:**
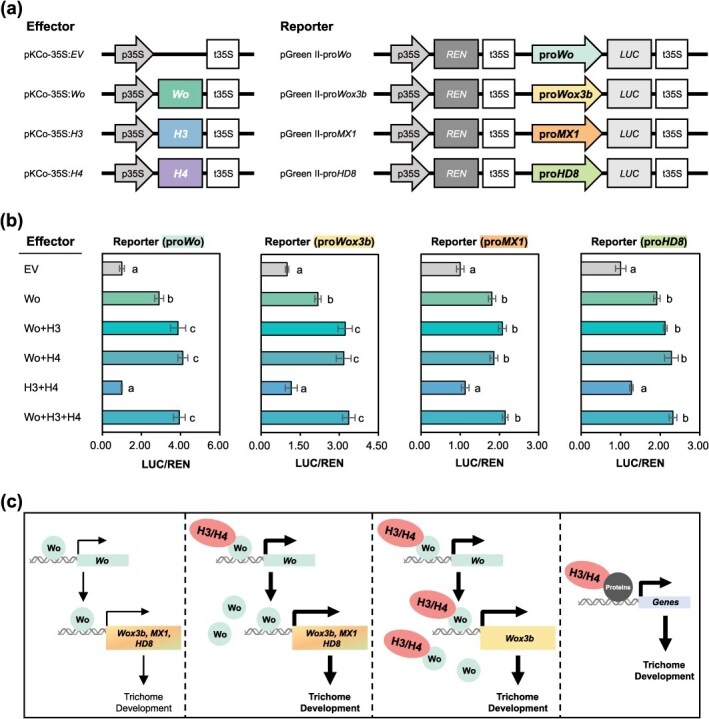
H3/H4-Wo protein complexes enhance the transcriptional activity of *Wo* and *Wox3b* genes. (a) Schematic overview of vector constructs used in DLR assays. The promoters of *Wo*, *Wox3b*, and *MX1* were cloned upstream of the LUC gene in the pGreen II-0800-LUC reporter vector (pGreen II-pro*Wo*, pGreen II-pro*Wox3b*, and pGreen II-pro*MX1*). Full-length *Wo*, *H3*, and *H4* genes were fused downstream of the 35S promoter in the pKCo effector vector (35S:*Wo*, 35S:*H3,* and 35S:*H4*). These reporter and effector constructs were coinfiltrated in tobacco leaves via agroinfiltration. An empty pKCo vector (35S:EV) served as a negative control. DLR assays were conducted 2 days postinfiltration. (b) Transactivation of *Wo*, *Wox3b*, *MX1*, and *HD8* promoters by H3/H4-Wo complexes. LUC and Renilla luciferase (REN) activities were measured, with 35S:EV serving as a negative control. LUC activity was calculated as the ratio of LUC to REN and normalized to that of the control. Data are presented as the mean (±SE) of eight biological replicates. Different letters indicate statistically significant differences (*P* < 0.05, one-way ANOVA with Tukey’s *post hoc* test). (c) Proposed model of H3/H4-Wo protein complexes in tomato trichome development. Left: Wo protein, synthesized via its own transcription, directly regulates the expression of trichome development-related genes, including *Wox3b*, *MX1*, and *HD8*. Center-left: H3 and H4 proteins interact with Wo to enhance *Wo* expression, thereby promoting *Wox3b*, *MX1*, and *HD8* expression. Center-right: More H3/H4-Wo complexes bind to the *Wox3b* promoter, further enhancing *Wox3b* expression. Right: H3 and H4 proteins may interact with other regulatory proteins, potentially modulating the expression of other trichome development genes.

We then performed DLR assays to investigate the roles of H3 and H4 in Wo-mediated transcriptional activity. Coexpression of Wo with H3, H4, or both resulted in a significant increase in Firefly luciferase (LUC) activity compared with Wo alone (Fig. 6a and b). Given that *Wo, Wox3b*, *MX1*, and *HD8* are direct targets of Wo ([27]; Fig. S19), we further explored the regulatory effects of H3 and H4 on these genes in combination with Wo. Compared with Wo expression alone, coexpression of Wo with H3, H4, or both resulted in a substantial increase in LUC activity driven by the *Wo*, *Wox3b* promoter. In contrast, no significant increase in LUC activity was observed for the *MX1* or *HD8* promoter under the same experimental conditions (Fig. 6a and b). This implies that H3 and H4 proteins are crucial for trichome development by differentially modulating Wo-mediated transcriptional regulators, such as Wo, Wox3b, MX1, and HD8.

## Discussion

ZFPs play important roles in various biological functions in eukaryotes by regulating gene expression or interacting with other proteins. They can be categorized into distinct groups based on the configuration of the cysteine and histidine residues, such as C2C2, C2H2, and C2HC. Among these, C2H2 ZFPs are the most abundant and specific to plants [34]. C2H2 ZFPs participate in multiple phases of plant development, from seed germination to fruit ripening [35]. Here, we established that H3 and H4 regulate multicellular trichome formation in tomato. The markedly higher expression of *H3* and *H4* in the trichome-enriched epidermal stem relative to the negligible levels observed in peeled-off stem tissue strongly suggests that these genes are involved in trichome function (Fig. 1c). Functional analysis of *h3* sko and *h4* sko mutants exhibited normal trichome development, whereas the *h3/h4* dko mutant had fewer glandular trichomes and more nonglandular trichomes (Fig. 2 and Fig. S3), implying redundant regulatory roles for *H3* and *H4*.

*H3* and *H4* regulated trichome development in diverse tissues, such as the leaves, stems, and hypocotyls. Phenotypic analysis of *Arabidopsis* trichome mutants with impaired *ZFP* genes, such as *gis*, *gis2*, and *zfp8*, revealed trichome-specific effects without noticeable impacts on other developmental aspects [11, 12]. Similarly, *h* and *h2* mutants exhibited trichome-specific phenotypes in tomato [22, 23]. In our study, *h3* sko and *h4* sko mutants closely resembled WT plants, except for the *h4* sko mutant, which showed reduced growth ability (Fig. S8a and b). Notably, the *h3/h4* dko mutant showed several distinct phenotypic changes compared to WT plants, including altered trichome density, shorter Type I trichomes (Fig. 2), and reduced leaf and overall plant size (Fig. S8c–f). Considering the extensive literature indicating the involvement of *ZFPs* in various aspects of plant development, our findings suggest that *H3* and *H4* are integral to trichome development and play essential roles in plant growth. Our findings indicate that *h3/h4* dko mutants have fewer glandular trichomes and more nonglandular trichomes, indicating that *H3* and *H4* positively regulate glandular trichomes but negatively influence nonglandular trichomes. However, the specific mechanisms by which *H3* and *H4* regulate these trichome types remain to be investigated. The *h* sko mutant had lower Type I trichome density and greater Type III and V density on the stem (Fig. 2 and Fig. S16; [25]), while all trichome types showed decreased density in the *wo* sko mutant (Fig. S12). The observed similarities and differences in trichome phenotypes among these mutants suggest that the development of each trichome type in tomato is governed by complex genetic pathways and distinct regulatory mechanisms.

The transcriptional regulation of *ZFP* genes during trichome development has been extensively studied in *Arabidopsis*. Prior studies have demonstrated hierarchical interactions, with *GIS3* regulating *GIS* and *GIS2* [13], *ZFP5* regulating *ZFP8* [1,4], and *ZFP6* controlling *ZFP5* [15]. These findings indicate a clear hierarchy among *ZFP* genes in the regulation of trichome development in *Arabidopsis*. In tobacco, *NbGIS* plays key roles in trichome development [36]; however, the involvement of other *ZFP* genes and their potential regulatory interactions remain largely unexplored. Our transcriptomic and qRT-PCR results provide new insights into these regulatory pathways. Specifically, we observed that *H3* and *H4* regulated *H* complementarily, but not *H2*, and that *H* did not regulate *H3* or *H4* (Fig. 4 and Fig. S18a). As H3 and H4 did not directly regulate *H* (Fig. S18b–d), it is likely that additional regulatory factors participate in this process. These findings suggest the presence of a complex and multifaceted transcriptional regulatory network governing trichome development in both tomato and Arabidopsis, with *ZFP* genes playing key roles. Further studies are needed to elucidate the additional regulatory components and interactions within this pathway.

Our study revealed that *HD8* expression, which is known to regulate trichome length in tomato [20, 21], was reduced in both *h3/h4* dko and *h4* sko plants. However, only the *h3/h4* dko mutant displayed a reduction in Type I trichome length, while the *h4* sko mutant did not show this phenotype, suggesting potential redundancy between *H3* and *H4* in trichome length regulation. Additionally, *wo* sko plants also showed reduced *HD8* expression, indicating that *H3*, *H4*, and *Wo* may collectively influence trichome length through *HD8* modulation. Y1H and DLR analyses confirmed that Wo directly regulates *HD8* expression while H3 and H4 do not (Fig. S19). After their formation, trichomes undergo morphogenesis to develop their shape, involving various genes associated with actin polymerization. For example, mutations in *Hairless* (*Hl*), *Hl-2*, and *Hl-3*, which encode SRA1, NAP1, and ARPC1, respectively, resulted in swollen and distorted trichomes due to disrupted actin polymerization [30, 37]. Previous studies have reported differing effects of *HD8* on trichome morphology. Xie *et al*. [21] observed distorted trichomes in *HD8* knockdown mutants, while Hua *et al*. [20] reported that *HD8* ko retained normal trichome morphology with reduced length. Our observations of *h3/h4* dko mutants align with the findings of Hua *et al*. [20], suggesting that *H3* and *H4* are involved specifically in trichome length regulation rather than morphogenesis. The differences observed between the knockdown and ko studies may reflect environmental influences, such as growth conditions, given the sensitivity of trichomes as the outermost epidermal cells to external factors. However, the precise reasons for these variations remain unclear and warrant further investigation.

Recent advances have broadened our understanding of the role of Wo and its regulated genes in tomato trichome development. Wo has been identified as a dose-dependent regulator of multicellular trichome fate because it regulates genes such as *MX1*, *Wox3B*, and *LFS* [27]. Additionally, Wo influences multicellular trichome morphogenesis by modulating the expression of *MX1*, *Wox3B*, and *BRANCHED2a* [38]. Recent findings have also suggested that trichomes serve as mechanosensors and highlight the involvement of Wo and MYC1 in terpene biosynthesis through signal transduction mechanisms [39]. Interestingly, Wo and its homologs HD7 and HD7L are implicated in the formation of interlocking trichomes and regulation of style length, which are crucial for self-pollination in tomato [40]. However, despite these findings, the mechanisms governing the regulation of the *Wo* gene itself remain largely unexplored. In our study, *Wo* and its direct target genes *Wox3b*, *MX1*, and *HD8* were downregulated in *h3/h4* dko plants, suggesting that *H3* and *H4* participate in the early stages of trichome initiation and elongation, likely by regulating *Wo*. Interestingly, the Y1H assay indicated that H3 and H4 do not directly bind to the *Wo* promoter (Fig. 4e), suggesting an indirect regulatory influence on *Wo*. Additionally, while the Y2H assay indicated autoactivity for H and H2, it showed no autoactivity for H3 and H4 (Fig. 3a). This suggests that unlike H and H2, which may function as transcription factors, H3 and H4 likely act as transcriptional regulators through alternative mechanisms. ZFP proteins, known for their capacity to form dimers, bind DNA, and interact with other proteins [41, 42], may provide one such mechanism. Despite the established roles of ZFPs in protein–protein interactions (PPIs), only few studies have considered the contributions of ZFP proteins in trichome development via dimerization. Our findings revealed that H3 and H4 form both homo- and heterodimers but do not engage in protein–protein interactions with H or H2 (Fig. 3). This observation suggests a distinct regulatory mechanism for trichome development between H3/H4 and H/H2. The ability of H3 and H4 to interact with each other may also explain their functional redundancy in promoting trichome formation and elongation. Additionally, these interactions likely play a crucial role in modulating the transcriptional regulatory network that governs trichome development, highlighting the importance of H3/H4 dimerization in orchestrating this complex process.

Similar to the well-characterized MBW complex in Arabidopsis, ZFP-Wo complexes may control trichome development in tomato by regulating the expression of downstream genes, although direct evidence for this hypothesis has been lacking. Our findings provide key insights into this regulatory mechanism by demonstrating that H3 and H4 interact with Wo (Fig. 5). Notably, these ZFP-Wo complexes enhanced the levels of *Wo* along with multiple trichome-related genes (Fig. 6a and b). This represents the first evidence on the direct regulatory role of ZFP-Wo complexes in trichome development. Notably, these complexes exerted differential regulatory effects on the direct target genes of Wo. While *Wox3b* expression was upregulated by the ZFP-Wo complexes, *MX1* and *HD8* expression was not (Fig. 6a and b), suggesting that the regulatory mechanisms may vary among target genes. Based on these findings, we hypothesized that ZFP-Wo protein complexes fine-tune the expression of Wo-dependent genes involved in trichome development in tomato (Fig. 6c). For instance, Wo regulates its own expression, directly influencing the expression of *Wox3b*, *MX1*, and *HD8* (Fig. 6c, left; [27]). H3/H4-Wo protein complexes further upregulate *Wo* gene expression, leading to increased levels of Wo protein and, consequently, enhancing the expression of *Wox3b*, *MX1*, and HD8 (Fig. 6c, center-left). Additionally, these complexes directly upregulate *Wox3b* expression (Fig. 6c, center-right). Considering the role of H3 and H4 in trichome gene regulation through their interaction with Wo, it is plausible that other proteins interacting with ZFPs also contribute to trichome development (Fig. 6c, right). Identifying these interacting proteins and elucidating their specific roles in trichome formation are of particular interest. Further investigations into how these protein complexes coordinate trichome development will provide important insights into the regulatory networks governing multicellular trichome formation in plants.

## Materials and methods

### Phylogenetic tree construction, sequence alignments, and protein motif predictions

Homologs corresponding to SlH (Solyc10g078970) and SlH2 (Solyc10g078990) in both tomato and *Arabidopsis* were identified using BLASTp searches in the Sol Genomics Network and protein BLAST searches of the National Center for Biotechnology Information. The retrieved amino acid sequences were aligned using ClustalW, and phylogenetic analysis was performed with MEGA7 using the neighbor-joining method and 1000-replicate bootstrap tests. To predict the motifs of the putative proteins H3 (Solyc03g058160) and H4 (Solyc10g080600), MEME Suite (http://meme-suite.org/) and LOCALIZER (http://localizer.csiro.au) were used.

### Plant materials and growth conditions

*Solanum lycopersicum* cv. Ailsa Craig (LA2838A) was used as the WT plant for all experiments, except for *wo* ko plants, where cv. M82 (LA3475) was used instead. Tomato seed germination and growth were conducted as previously described [23]. Tobacco (*Nicotiana benthamiana*) seeds were sown in 11-cm-diameter pots filled with a soil mixture and grown in a growth chamber at 25°C and 60% humidity under a 16-h light/8-h dark photoperiod. Agroinfiltration was performed on 5-week-old plants.

### Subcellular localization

H3 and H4 proteins were fused with a YFP tag. PCR amplification of *H3* and *H4* lacking a stop codon was performed using cDNA and H3-local and H4-local primer sets, respectively (Table S3). The resulting H3 and H4 fragments were incorporated into the *BamHI* and *XhoI* sites within the pENTR3C entry vector (#A10464, Invitrogen, Carlsbad, CA, USA). The genes within the entry vectors were translocated to pBCo-DC-YFP destination vectors using a Gateway LR reaction system (#11791–020, Invitrogen). The resulting vectors were designated pBCo-H3:YFP and pBCo-H4:YFP. As a positive control, MYC2 fused with a cyan fluorescent protein (CFP) tag in the pBCo vector (pBCo-MYC2:CFP) was used [43]. Agroinfiltration of tobacco leaves and visualization of the YFP and CFP signals were conducted as previously described [23].

### Total RNA isolation and qRT-PCR

To examine tissue-specific expression of *H3* and *H4*, samples were collected from 6-week-old WT plants, including the third compound leaves from the shoot apical meristem, immature floral buds (0.8 cm in length), fully opened flowers (2 cm in length), and roots (5 cm above the root apical meristem), whole stems located between the second and third compound leaves, stem epidermal layers, and peeled-off stems (both from the same stem section). All samples were immediately frozen in liquid nitrogen. For trichome collection, stems between the second and third compound leaves of 6-week-old WT plants were cut and frozen in liquid nitrogen. Using a frozen flat-end spatula, the stem surface was gently scraped to isolate trichomes. To assess the expression of trichome-related genes, corresponding leaf tissues were collected from both WT and transgenic plants at 6 weeks. Total RNA isolation, cDNA synthesis, and qRT-PCR were performed according to established protocols [23]. *SlACT7* (Solyc03g078400) served as the internal standard for qRT-PCR. All primer sets are presented in Table S3.

### Plant transformation and selection of transgenic plants

To generate ko vectors for *H3* and *H4*, specific sequences for single-guide RNA (sgRNA) were designed using CRISPR RGEN Tools (http://rgenome.ibs.re.kr). Primer sets for the *H3* and *H4* sko (H3-sko-sgRNA and H4-sko-sgRNA) and dko sgRNA (H3-dko-sgRNA and H4-dko-sgRNA) are presented in Table S3. H3-sko-sgRNA and H4-sko-sgRNA were incorporated into the pHAtC binary vector [31]. H3-dko-sgRNA and H4-dko-sgRNA were cloned into the sgRNA1 and sgRNA2 sites of the pAGM4723 vector, respectively [32]. The resulting ko vectors were transformed into *Agrobacterium tumefaciens* strain LBA4404 using cotyledon explants from WT plants as previously described [30]. Genomic DNA (gDNA) regions encompassing sgRNA sequences were amplified using h3-ko-sel or h4-ko-sel primer sets (Table S3) to confirm mutations in the *H3* or *H4* genes of transgenic plants. The PCR products were sequenced by Macrogen Co. Ltd. (Seoul, Republic of Korea). Homozygous lines for *h3* sko, *h4* sko, and *h3/h4* dko T_0_ were selected and cultivated to obtain T_1_ seeds. *Cas9* detection in T_1_ plants involved the PCR amplification of gDNA from eight plants for each ko line using the Cas9-sko-sel and Cas9-dko-sel primer sets (Table S3). *Cas9*-free T_1_ plants were selected, and homozygous *h3* ko or *h4* ko plants were confirmed via direct sequencing. *Cas9*-free homozygous *h3* sko, *h4* sko, and *h3/h4* dko T_2_ lines were selected for subsequent experiments. The Supplementary Text discusses the plant transformation and construction of ko vectors for *Wo* and *H*.

### Microscopic observation of trichome phenotype

Trichome phenotypes were observed using a dissecting microscope (CH-M205A, Leica Microsystems, Wetzlar, Germany) and a cryo-scanning electron microscope (CryoSEM, Hitachi High-Tech Corporation, Tokyo, Japan), following previously established protocols [44]. Six-week-old WT and transgenic plants were used to compare trichome density and length. Regions of leaves and stems for microscopic observation were selected based on previously described methods [23]. Trichome density was calculated by counting the total number of trichomes within a 5 mm^2^ area on the surfaces of leaves and stems, and trichome length was measured at the leaf margins and stem edges. Microscopic observations were also performed on sepals of immature floral buds (0.8 cm in length) and the hypocotyl (1 cm below the cotyledon).

### RNA-sequencing analysis

To explore the genes controlled by *H3* and *H4*, total RNA samples were isolated from the third compound leaves from the shoot apical meristem of 6-week-old WT and *h3/h4* dko plants using TRIzol reagent (#15596018, Invitrogen). Three of the third compound leaves were pooled as one biological replicate, and three biological replicates were prepared for each line. The integrity and quantity of the total RNA were assessed using agarose gel electrophoresis and a NanoPhotometer (NP80, Implen, München, Germany). RNA-Seq was performed by SEEDERS Inc. (Seoul, Republic of Korea). RNA quality checks and library preparation were conducted as previously described [45]. Sequencing and sequence preprocessing were performed according to established protocols [46]. Mapping was performed as previously described [47]. DEGs were identified based on a log_2_FoldChange ≥ 1.0 and false discovery rate (FDR) ≤ 0.01. The FDR was used to determine the *P*-value threshold in binomial tests and was calculated using DESeq software [48]. The DEGs were subjected to GO term enrichment analysis using ShinyGo 0.80 (http://bioinformatics.sdstate.edu/go/). The levels of trichome-related genes in the RNA-Seq data were visualized using heat maps in SRplot [49].

### Y1H assay

To investigate the potential binding of H3 and H4 to the *Wo* and *H* promoters, Y1H experiments were conducted using a Matchmaker Gold One-Hybrid System (K1603–1, Clontech Laboratories, Mountain View, CA, USA). The full-length coding sequences (CDSs) of Wo, H3, and H4 were cloned into the *NdeI* and *BamHI* sites of the pGADT7 prey vector (pGADT7-Wo, H3 pGADT7-H3, and pGADT7-H4, respectively). The promoter regions of *Wo* (2293 bp), *H* (2,731 bp), and *HD8* (3334 bp) were PCR-amplified from gDNA using specific primer sets (pro*Wo*-Y1H, pro*H*-Y1H, and pro*HD8*-Y1H, respectively; Table S3). The *Wo* and *HD8* promoter fragments were inserted into the pLacZi bait vector at the *Kpn*I and *Xho*I sites (pLacZi-pro*Wo* and pLacZi-pro*HD8*). The *H* promoter fragment was cloned into the pLacZi bait vector at the *Xma*I and *Sal*I sites (pLacZi-pro*H*). Each bait vector was digested with *ApaI*, and the linearized bait vectors were cotransformed into *Saccharomyces cerevisiae* strain YM4271 along with the prey vectors (pGADT7-Wo, pGADT7-H3, or pGADT7-H4). Yeast colonies containing each bait vector and pGADT7-EV were used as negative controls. Transformed yeast cells were cultured on an SD/-Ura/-Leu medium at 30°C for 3 days. Colonies were then transferred to SD/-Ura/-Leu plates containing X-gal (80 mg/l) for *in vivo* X-gal plate assays. For the colony-lift filter assay, yeast colonies grown on SD/-Ura/-Leu medium were transferred to filter paper, frozen, and thawed twice with liquid nitrogen. After this process, the filter paper with colonies was placed on another filter soaked in Z-buffer/X-Gal solution. The colonies were then incubated at 30°C for 5 min to 4 h following the protocol outlined in the Yeast Protocols Handbook (Takara Bio, PT3024–1) to observe any color change indicating potential protein-DNA interactions.

### DLR assay

DLR assays were conducted to validate promoter activity. The full-length CDSs of *H3*, *H4*, and *Wo* were PCR-amplified using cDNA and the primers presented in Table S3. The resulting amplicons were inserted into the pENTR3C entry vector (#A10464, Invitrogen) and transferred to the pKCo-DC destination vector using the Gateway LR reaction system (#11791–020, Invitrogen). The constructed effector vectors were designated pKCo-35S:H3, pKCo-35S:H4, and pKCo-35S:Wo; pKCo-35S:EV served as a negative control. The promoter regions of *Wo* (2293 bp), *H* (2,731 bp), *Wox3b* (2503 bp), *MX1* (3320 bp), and *HD8* (3334 bp) were amplified from the gDNA using the primers presented in Table S3. Each amplified promoter fragment was inserted into the pGreen II-LUC reporter vector and named pGreen II-pro*Wo*, pGreen II-pro*H*, pGreen II-pro*Wox3b*, pGreen II-pro*MX1*, and pGreen II-pro*HD8*. The effector vectors, reporter vectors with pSoup helper vectors [50], and pCAMBIA-p19 helper vectors were individually transformed into *A. tumefaciens* strain GV3101. After culturing until an OD_600_ of 0.5, equal amounts of effector, reporter, and p19 mixtures were infiltrated in *N. benthamiana* leaves. After 2 days of incubation, LUC and REN luciferase activities were measured using a Dual-Luciferase Reporter Assay System (E1910, Promega, Madison, WI, USA) and quantified with a microplate reader (Spark, Tecan, Männedorf, Switzerland), following the manufacturer’s instructions.

### Y2H assay

To test the autoactivation activities of H, H2, H3, and H4, Y2H assays were performed using a Matchmaker GAL4 Two-Hybrid System 3 (Clontech). The CDSs of *H*, *H2*, *H3*, and *H4* were PCR-amplified using cDNA and the primers in Table S3. The amplified fragments were cloned into the pGBKT7 bait vector, generating plasmids pGBKT7-H, pGBKT7-H2, pGBKT7-H3, and pGBKT7-H4. Each plasmid was cotransformed into *S. cerevisiae* strain AH109 with pGADT7-EV to test for autoactivation activity. Positive controls included yeast colonies containing pGBKT7–53 and pGADT7-T, whereas negative controls included pGBKT7-EV and pGADT7-EV. To investigate whether H3 and H4 form homo- or heterodimers, the CDSs of *H*, *H2*, *H3*, and *H4* were cloned into the pGADT7 prey vector, resulting in pGADT7-H, pGADT7-H2, pGADT7-H3, and pGADT7-H4, respectively. Then, pGBKT7-H3 or pGBKT7-H4 was cotransformed into yeast cells harboring pGADT7-H, pGADT7-H2, pGADT7-H3, or pGADT7-H4, respectively. To examine the interactions of H3 and H4 with Wo or CycB2, a previously cloned pGADT7 vector containing the CDS of *Wo* or *CycB2* was used [23]. pGADT7-Wo or pGADT7-CycB2 was cotransformed into yeast cells with pGBKT7-H3 or pGBKT7-H4. Transformed yeast cells were incubated on double (SD/-Trp/-Leu) or quadruple (SD/-Trp/-Leu/-His/-Ade) dropout medium at 30°C for 4 days.

### Preparation of recombinant proteins

The cDNAs of *H3* and *H4* were PCR-amplified and subcloned into the pGEX 4 T-1 (#27458001, Addgene, Watertown, MA, USA) and pCold TF (#3365, Takara Bio, Shiga, Japan) vectors using the primers presented in Table S3. For cloning into pGEX 4 T-1, a Strep tag (SAWRHPQFGG) was attached to the C-terminus for purification, as previously described [51]. *Escherichia coli* BL21-CodonPlus (DE3) cells harboring *H3*/pGEX 4 T-1 and *H4*/pGEX 4 T-1 constructs were used to express recombinant H3 and H4 proteins with GST and Strep tags at the N- and C-termini, respectively. DE3 cells harboring *H3*/pCold TF and *H4*/pCold TF constructs were also used to express H3 and H4 proteins with 6 × His and TF tags at the N-terminus, respectively. For protein expression, *E. coli* cells incubated at 37°C to an OD_600_ of 0.4–0.6 were further incubated at 20°C for pGEX 4 T-1 constructs or 15°C for pCold TF constructs for 1 h and then treated with IPTG (1 mM, final concentration). After overnight incubation, the cells were harvested and resuspended in ice-cold TE buffer (100 mM Tris, 1 mM EDTA; pH 8.0) or NPI buffer (50 mM NaH_2_PO_4_, 300 mM NaCl, 20 mM imidazole; pH 8.0). Protein extracts were obtained via sonication and centrifugation. The soluble extracts were filtered using a syringe filter (#431220, Corning, Corning, NY, USA), and the recombinant proteins (H3 and H4) were purified using a Strep-Tactin Sepharose (#2–1202-001, IBA Lifesciences, Göttingen, Germany) or Ni-NTA agarose (#30210, Qiagen, Hilden, Germany) column. Recombinant Wo protein containing Myc/His tags at the C-terminus was expressed and purified as previously described [23].

### Pull-down assay

Pull-down assays were conducted to verify homotypic and heterotypic interactions between H3 and H4 and investigate the PPIs between Wo and H3 or H4 and the interaction of Wo with H3 and H4 *in vitro*. Briefly, 2 μg of each protein was incubated in 500 μl of GST pull-down buffer (50 mM Tris–HCl [pH 7.5], 1 mM DTT, 150 mM NaCl, 0.6% Tween-20, and 100 μg/ml BSA) or His pull-down buffer (50 mM NaH_2_PO_4_ [pH 8.0], 300 mM NaCl, 10 mM Imidazole) at 4°C for 60 min, before being treated with 40 μl of glutathione resin or Ni-NTA agarose and further incubated for 60 min. After centrifugation, the proteins in the supernatant and precipitate were detected using Clarity Max Western ECL Substrate (#1705062, Bio-Rad Laboratories, Hercules, CA, USA) with TF-specific antibody (#M201, Takara Bio; 1:5000) for His/TF-fused H3 and H4, GST-specific antibody (sc-138, Santa Cruz Biotechnology, Dallas, TX, USA; 1:3000) for GST/strep-fused H3 and H4, and Myc-specific antibody (9B11, Cell Signaling Technology, Danvers, MA, USA; 1:1000) for Myc/His-fused Wo.

### Bimolecular fluorescence complementation assay

H3, H4, and Wo proteins were labeled with a YFP tag as follows: *H3* and *H4*, which lack a stop codon, were PCR-amplified using cDNA and the primers presented in Table S3. The resulting fragments were inserted into the pENTR3C entry vector (#A10464, Invitrogen), and the genes in the entry vectors were transferred to the pBA002-nEYFP-DC and pBA002-cEYFP-DC destination vectors using the Gateway LR reaction system (#11791–020, Invitrogen). The constructed vectors were named pBA002-nEYFP-H3, pBA002-cEYFP-H3, pBA002-nEYFP-H4, pBA002-cEYFP-H4, and pBA002-cEYFP-Wo. pBA002-cEYFP-EV was used as the negative control. The constructed BiFC vectors and pCAMBIA-p19 helper vectors were transformed into *A. tumefaciens* strain GV3101; *Agrobacterium* cells were cultured until an OD_600_ of 0.5, mixed equally (nEYFP, cEYFP, and p19), and infiltrated in *N. benthamiana* leaves. After 2 days of incubation, DAPI (1 μg/ml) was injected into the leaves 3 h before confocal microscopy observation. YFP and DAPI signals were visualized using a Leica SP8× gSTED confocal laser scanning microscope (Leica Microsystems) equipped with a 40× water immersion objective (Leica Microsystems). The excitation/emission wavelengths for YFP and DAPI were set at 514/520–570 nm and 405/410–470 nm, respectively.

## Supplementary Material

Web_Material_uhaf008

## Data Availability

All data are incorporated into the article and its online supplementary material.
