# Supplementary material for: SlH3 and SlH4 promote multicellular trichome formation and elongation by upregulating Woolly in tomato
Source: Hortic Res. 2025 Jan 24;12(4):uhaf008. doi: 10.1093/hr/uhaf008 (PMC11908826; doi:10.1093/hr/uhaf008)
Supplement: Web_Material_uhaf008 [file web_material_uhaf008.zip › H3_and_H4_MS_HR_supplementary information.docx]

**Supplementary Information**

**Materials and Methods**

***Plant transformation and selection of transgenic plants***

To construct ko vectors for *Wo* and *H*, specific sgRNA sequences were generated using CRISPR RGEN tools (http://rgenome.ibs.re.kr). The primer sets for *Wo* sko (Wo-sko-sgRNA) and *H* sko (H-sko-sgRNA) are presented in Table S3. Wo-sko-sgRNA and H-sko-sgRNA were cloned into the pAGM4723 and pHAtC binary vectors, respectively. Plant transformation was performed as previously described (Kang *et al.*, 2016). gDNA containing sgRNA sequences derived from *wo* sko or *h* sko T_0_ plants were amplified using the Wo-sko-sel and H-sko-sel primer sets (Table S3) to verify the mutations in the *Wo* and *H* genes, respectively. The Materials and Methods section of the main text outlines further procedures, including sequence analysis to confirm homozygosity in *wo* and *h* sko T_0_ plants, as well as the selection of *Cas9*-free lines in *wo* and *h* sko T_1_ plants.

**Reference**

**Kang J-H, Campos ML, Zemelis-Durfee S, Al-Haddad JM, Jones AD, Telewski FW, Brandizzi F, Howe GA. 2016.** Molecular cloning of the tomato *Hairless* gene implicates actin dynamics in trichome-mediated defense and mechanical properties of stem tissue. *Journal of Experimental Botany* **67**: 5313–5324.

**Supplementary Figures**

**
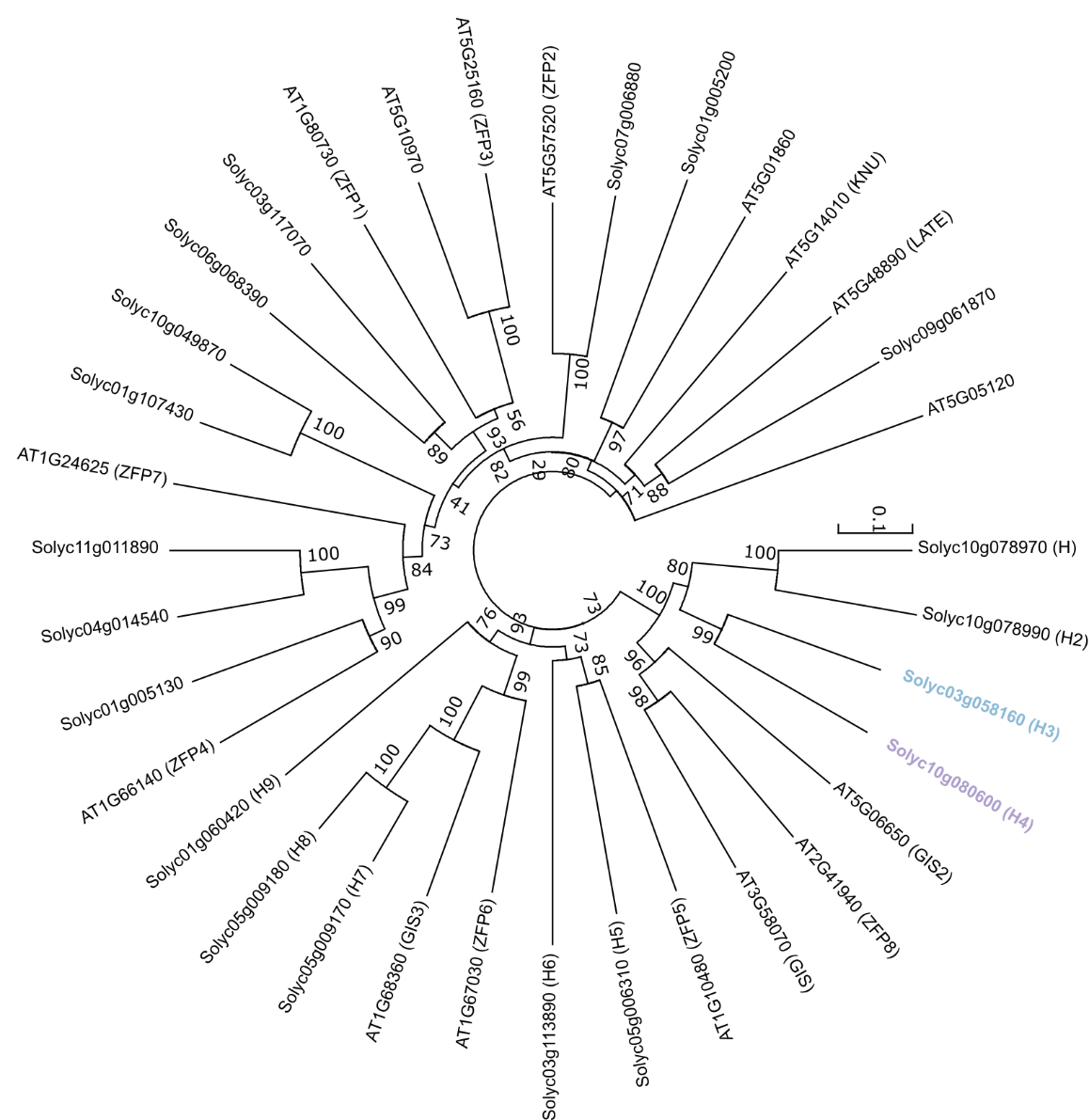
**

**Fig. S1. Phylogenetic analysis of SlH2 homologs in tomato and Arabidopsis.**

Phylogenetic analysis to identify homologs of H2 (Solyc10g078990) in tomato and Arabidopsis was conducted using the neighbor-joining method in MEGA7 based on amino acid sequences aligned using ClustalW. Bootstrap testing (1,000 replicates) provided the percentage values adjacent to the branches. The scale bar represents 0.1 amino acid substitutions per site. H3 (Solyc03g058160) and H4 (Solyc10g080600) are indicated in blue and purple, respectively.

**
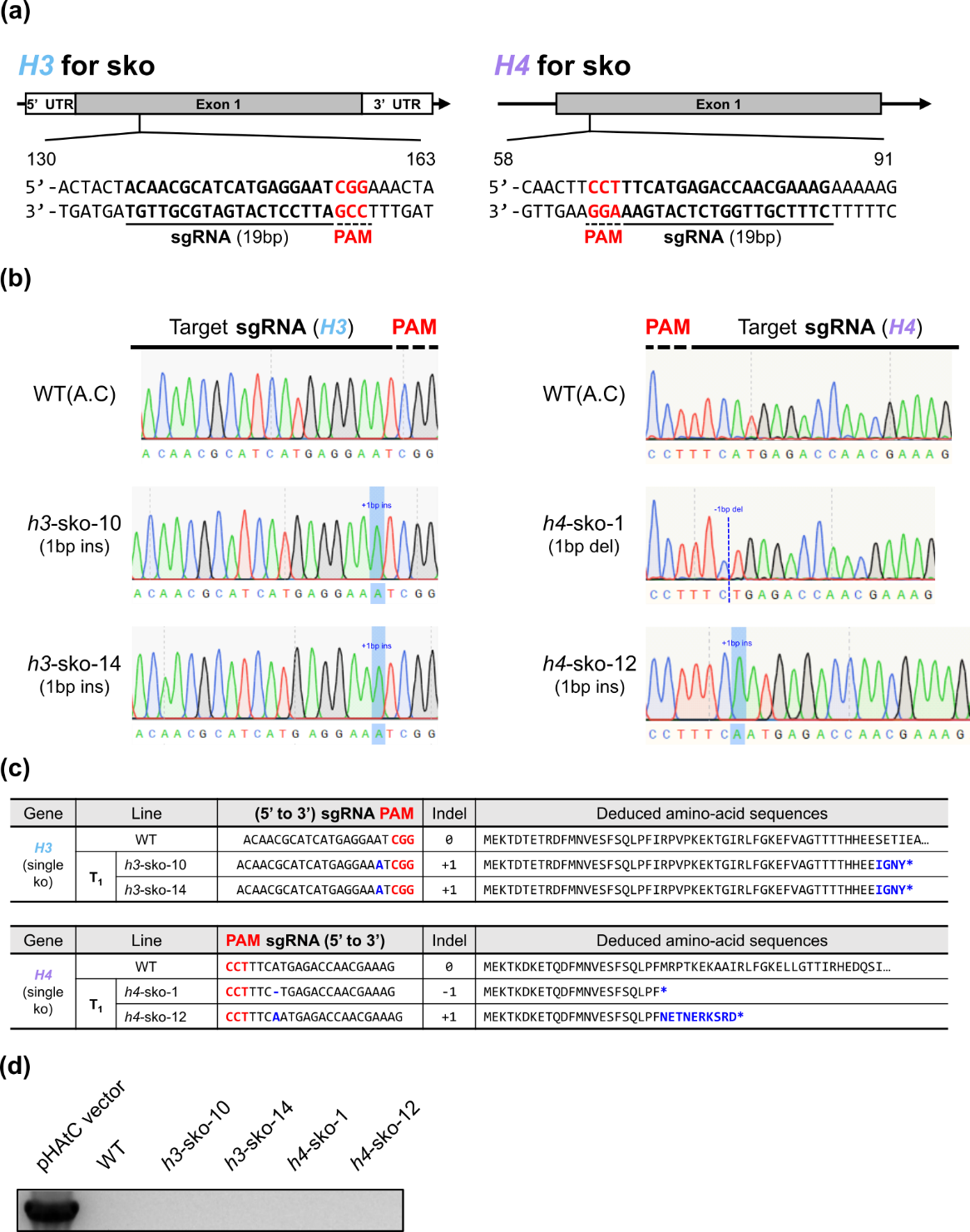
**

**Fig. S2. Development of *h3* sko and *h4* sko plants using the CRISPR-Cas9 system.**

(**a**) Gene structures and sgRNA target sites of *H3* and *H4* for generating *h3* sko and *h4* sko plants. The sgRNA sequences are underlined, and the PAM sequences (NGG) are indicated with dotted lines and red letters. (**b**) Sequence analysis of *h3* sko T_1_ and *h4* sko T_1_ plants. Modified nucleotides in *h3* sko and *h4* sko plants are highlighted with a blue background (insertion) or cursor (deletion). (**c**) Genotypes and deduced amino acid sequences of *h3* sko T_1_ (upper table) and *h4* sko T_1_ plants (lower table). The nucleotides colored in black and red represent the sgRNA and PAM sequences, respectively. Blue letters and hyphens in the nucleotide sequences of *h3* sko and *h4* sko plants indicate nucleotide insertions and deletions, respectively. Blue letters in the amino acid sequences of *h3* sko and *h4* sko plants indicate modified amino acid sequences compared to those of WT plants. Asterisks denote a premature stop codon. (**d**) Selection of transgene-free *h3* sko T_1_ and *h4* sko T_1_ lines. The agarose gel image indicates the presence or absence of the PCR-amplified *Cas9* gene from the gDNA of *h3* sko or *h4* sko plants. The pHAtC vector served as a positive control, while the gDNA of WT plants was used as a negative control.

**
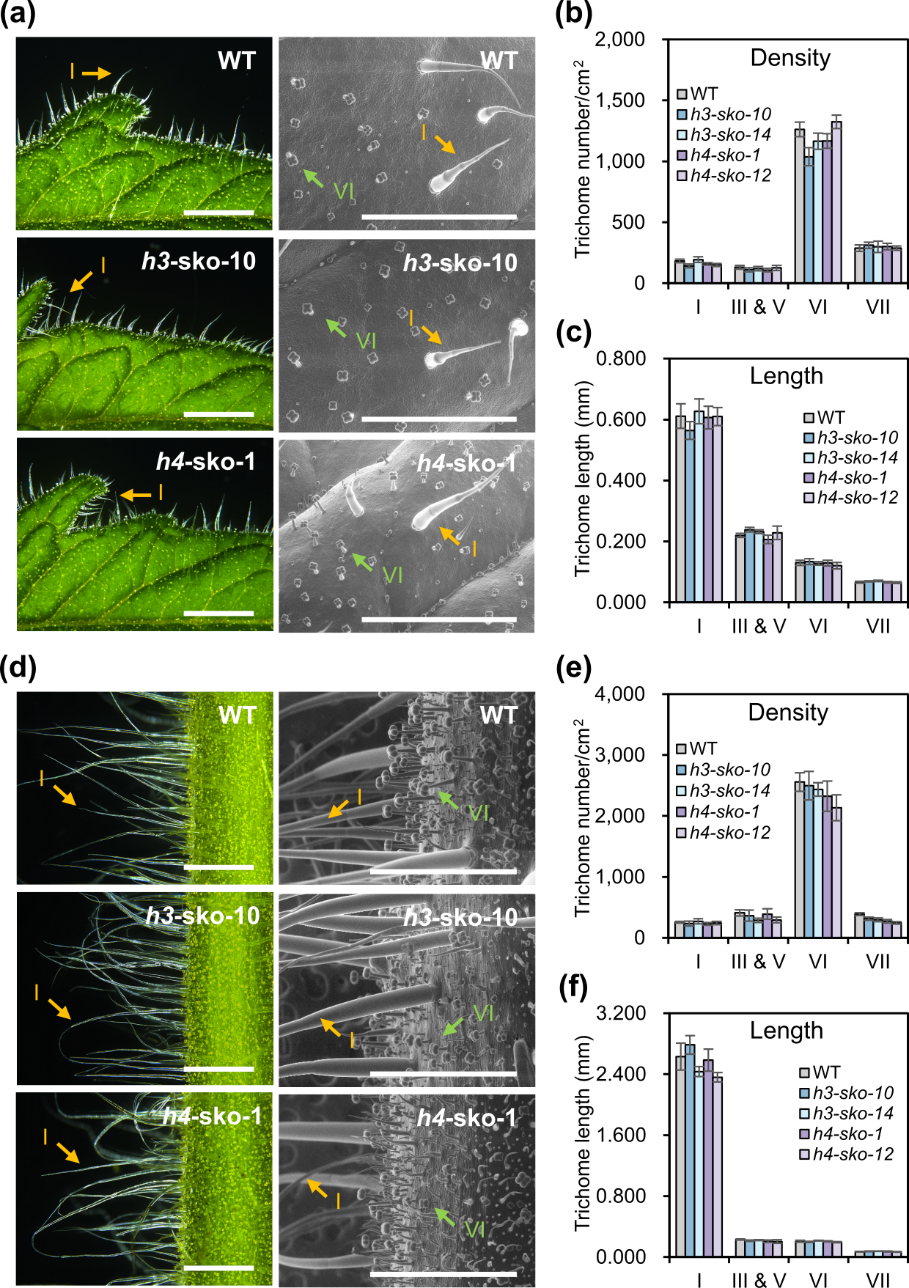
**

**Fig. S3. Trichome phenotypes of *h3* sko and *h4* sko plants.**

(**a**) Dissection microscopy (DM) and scanning electron microscopy (SEM) images of leaves in WT, *h3* sko, and *h4* sko plants. Scale bars: 2 mm (DM) and 1 mm (SEM). (**b, c**) Trichome density (b) and length (c) on leaves in WT, *h3* sko, and *h4* sko plants. (**d**) DM and SEM images of stems in WT, *h3* sko, and *h4* sko plants. Scale bars: 2 mm (DM) and 1 mm (SEM). (**e, f**) Trichome density (e) and length (f) on stems in WT, *h3* sko, and *h4* sko plants. All images were taken from six-week-old plants. Data are presented as the mean (±SE) of six biological replicates.


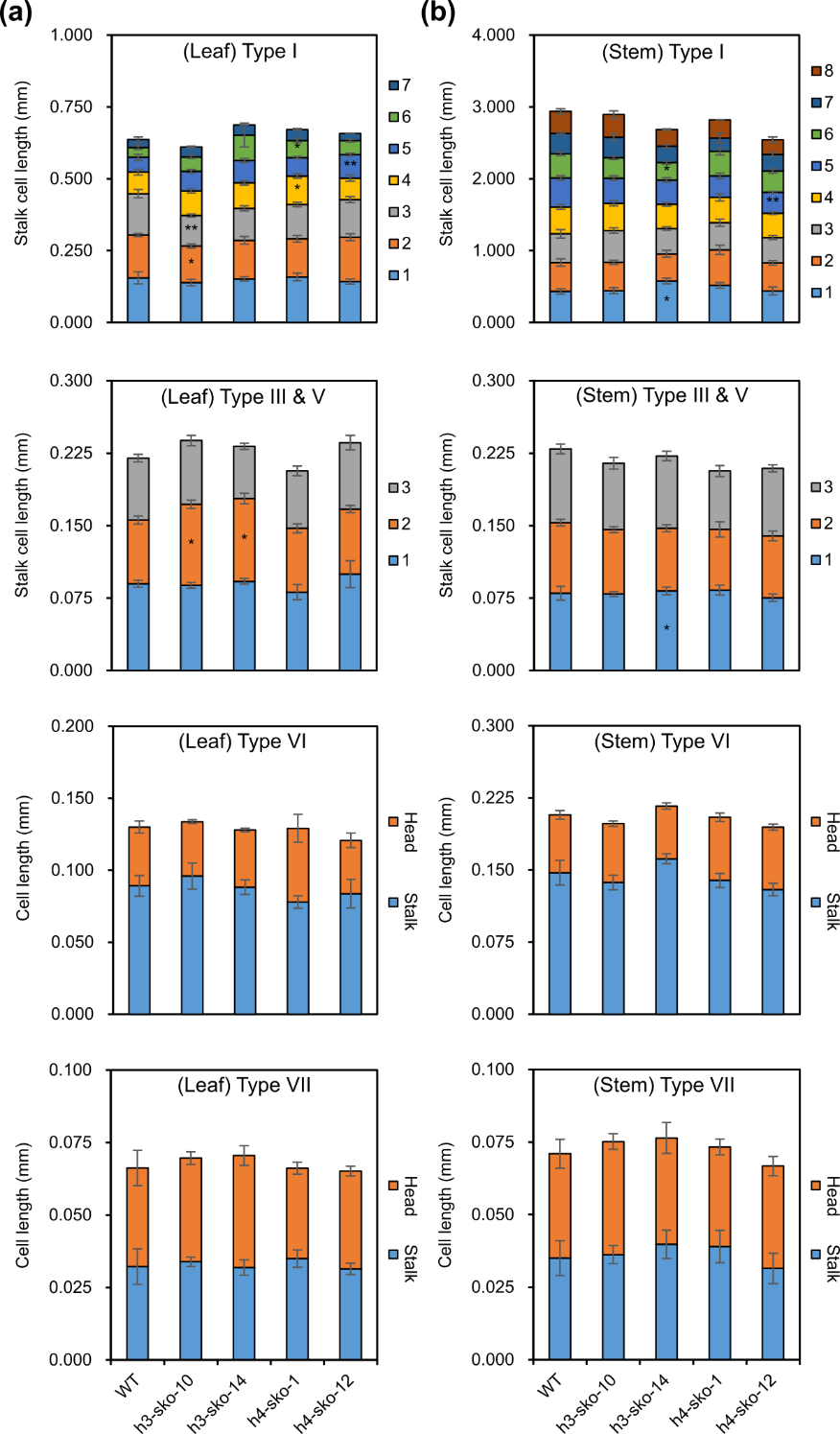


**Fig. S4. Analysis of trichome cell number and length in *h3* sko and *h4* sko plants.**

(**a, b**) Cell number and length of each cell type within trichomes on leaves (a) and stems (b) in WT, *h3* sko, and *h4* sko plants. Numbers on the right indicate stalk cell order from bottom (1) to top (7 or 8 for type I trichomes; 3 for type III and V trichomes). “Stalk” and “Head” refer to the stalk and glandular head cells of type VI and VII trichomes, respectively. Data are presented as the mean (±SE) of three biological replicates. Asterisks indicate significant differences between WT and sko plants (unpaired Student’s *t*-test: **P* < 0.05; ***P* < 0.01).

**
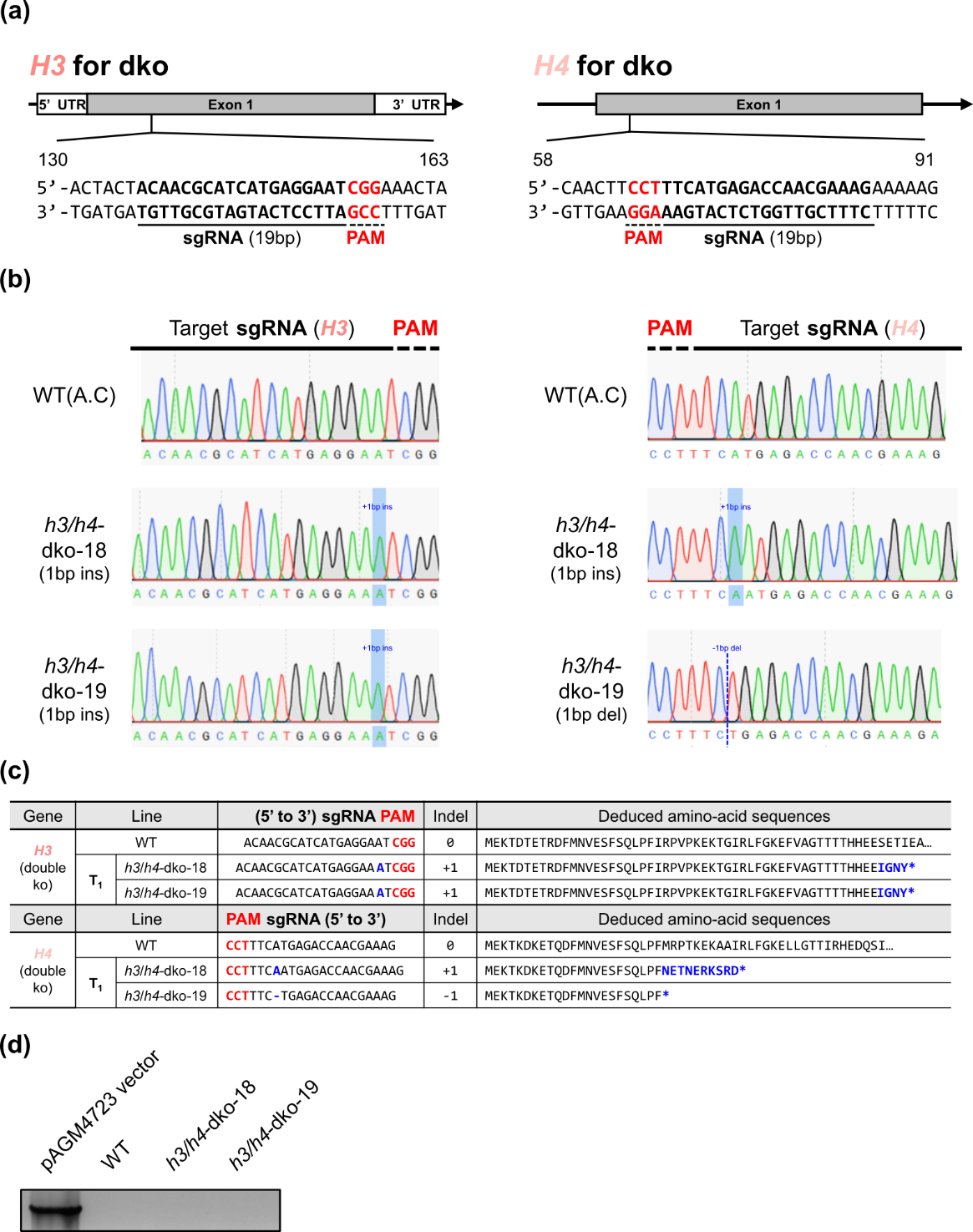
**

**Fig. S5. Development of *h3*/*h4* dko lines using the CRISPR-Cas9 system.**

(**a**) Gene structures and sgRNA target sites of *H3* and *H4* for generating *h3*/*h4* dko plants through the CRISPR-Cas9 system. The sgRNA and PAM sequences are marked in the same manner as in Fig. **S2a**. (**b**) Sequence analysis of *h3*/*h4* dko T_1_ plants. Modified nucleotides in the *h3*/*h4* dko plants are denoted in the same manner as in Fig. **S2b**. (**c**) Genotypes and deduced amino acid sequences of *H3* (upper side) and *H4* (upper side) in *h3*/*h4* dko T_1_ plants. Altered nucleotides and amino acids are presented in the same way as in Fig. **S2c**. (**d**) Selection of transgene-free *h3*/*h4* dko T_1_ lines. The agarose gel image illustrates the presence or absence of the PCR-amplified *Cas9* gene in the gDNA of *h3*/*h4* dko plants. The pAGM4723 vector served as a positive control, and the gDNA of WT plants was used as a negative control.

**
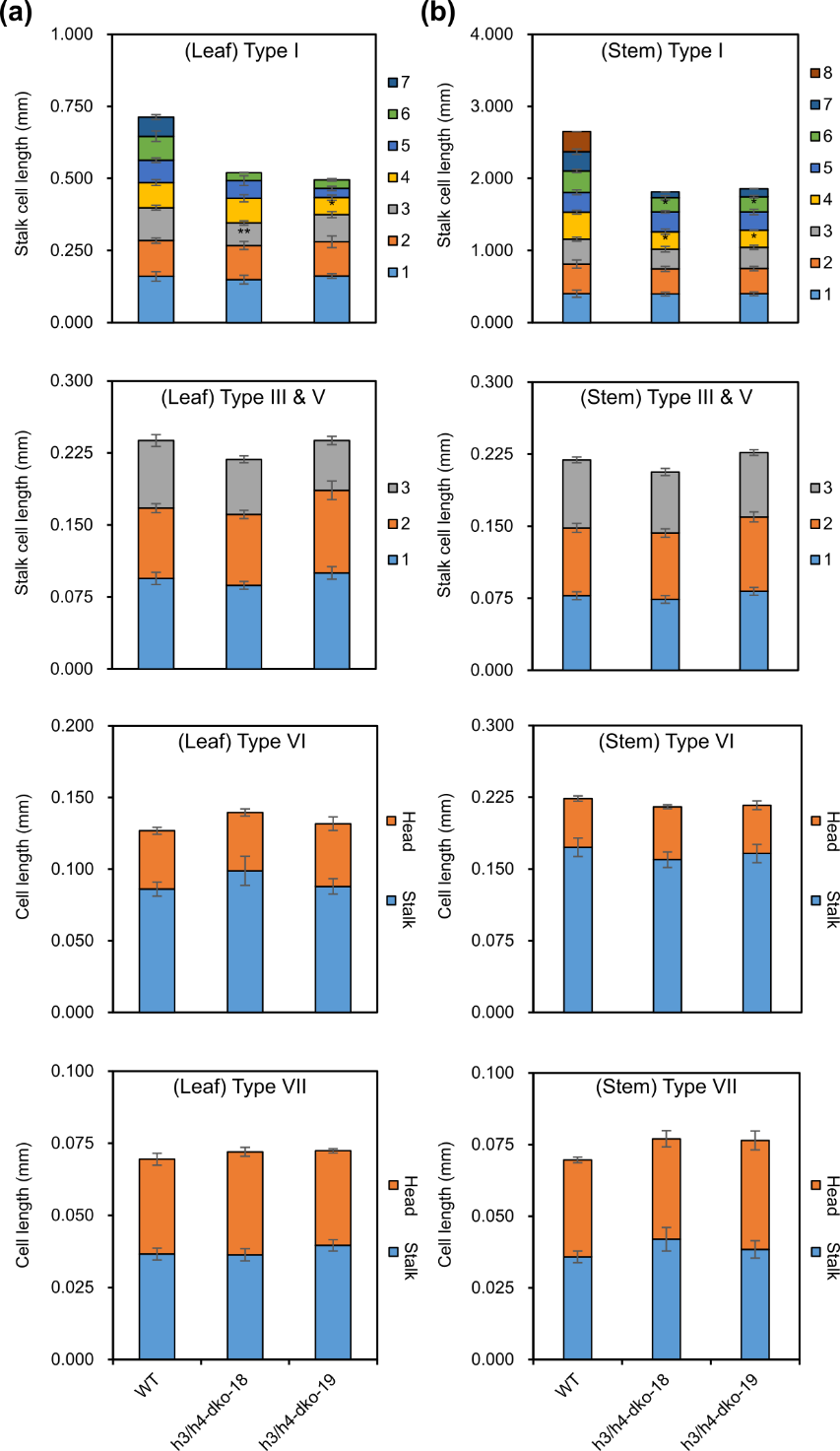
**

**Fig. S6. Analysis of trichome cell number and length in *h3/h4* dko plants.**

(**a, b**) Cell number and length of each cell type within trichomes on leaves (a) and stems (b) in WT and *h3/h4* dko plants. Numbers on the right indicate stalk cell order from bottom (1) to top (7 or 8 for type I trichomes; 3 for type III and V trichomes). “Stalk” and “Head” refer to the stalk and glandular head cells of type VI and VII trichomes, respectively. Data are presented as the mean (±SE) of three biological replicates. Asterisks indicate significant differences between WT and *h3/h4* dko plants (unpaired Student’s *t*-test: **P* < 0.05; ***P* < 0.01).

**
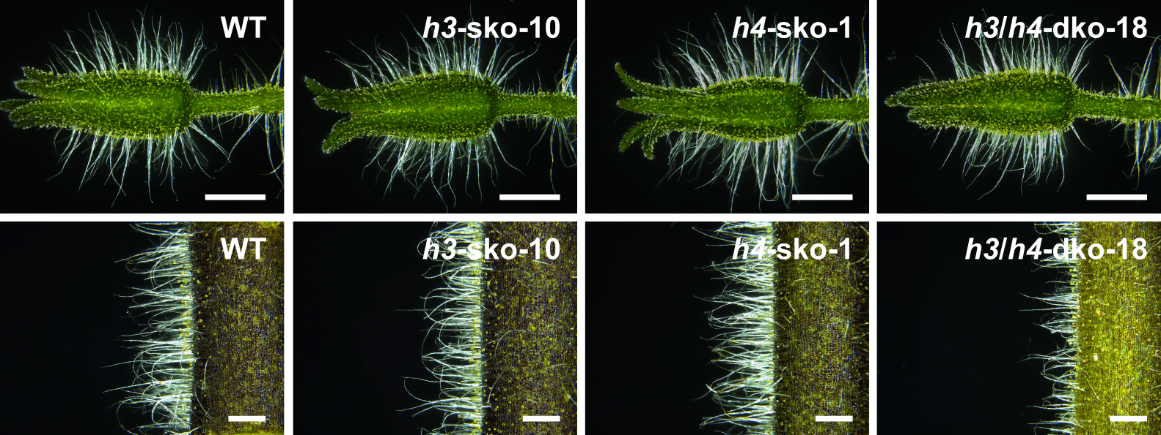
**

**Fig. S7. Trichome phenotypes on the sepals and hypocotyls of *h3* sko*, h4* sko, and *h3/h4* dko plants.**

Dissection micrographs of sepals are presented in the upper panel, whereas those of the hypocotyls are shown in the lower panel. Scale bar: 2 mm. All images were taken from 6-week-old plants.


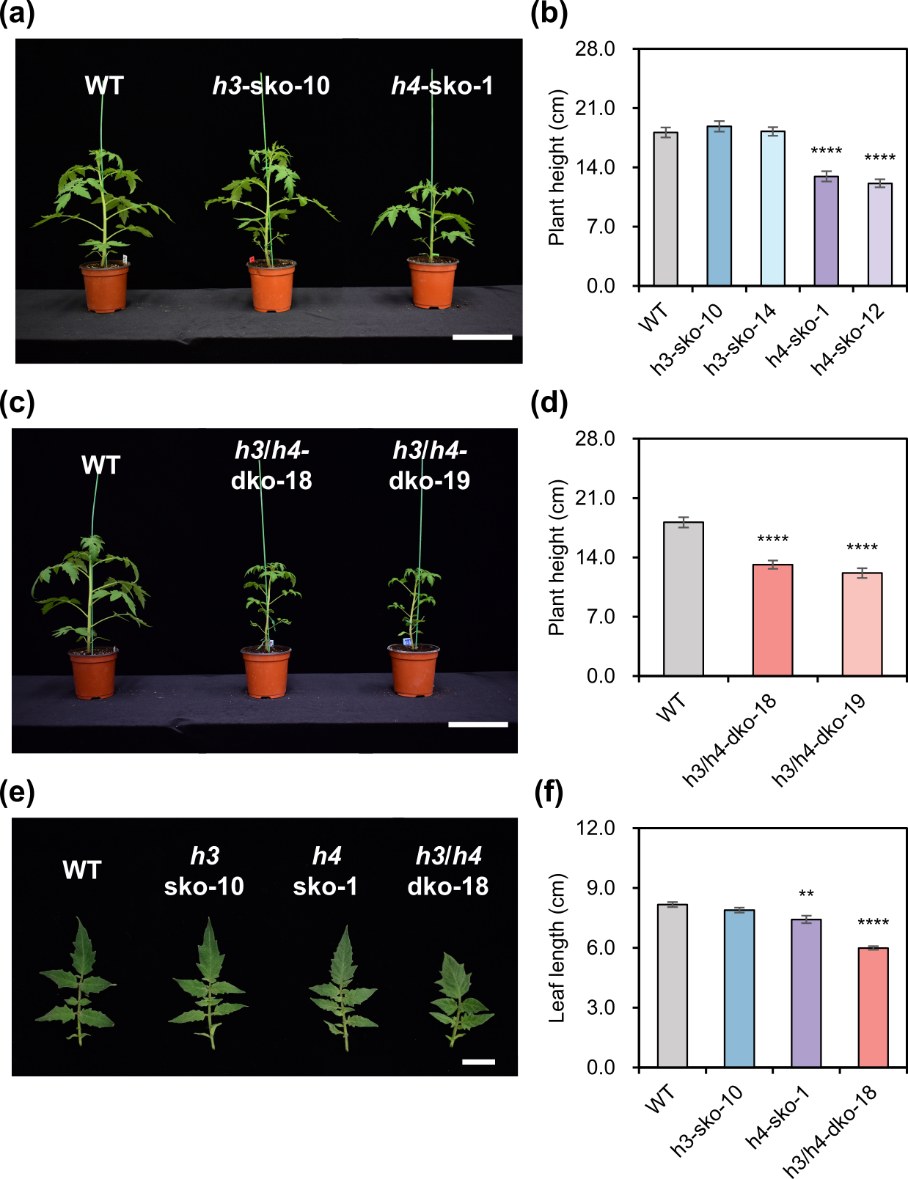


**Fig. S8. Growth of *h3* sko*, h4* sko, and *h3/h4* dko plants.**

(**a, c**) Photographs of 6-week-old WT, *h3* sko, and *h4* sko plants (a) and 6-week-old WT and *h3/h4* dko plants (c). Scale bar: 10 cm. (**b, d**) Heights of 6-week-old WT, *h3* sko, and *h4* sko plants (b) and 6-week-old WT and *h3/h4* dko plants (d). (**e, f**) Photograph (e) and lengths of compound leaves of 6-week-old WT and ko plants (f). Scale bar: 2 cm. Data in (b), (d), and (f) present the mean (±SE) of eight biological replicates. ***P* < 0.01, *****P* < 0.0001 (unpaired Student’s *t*-test).


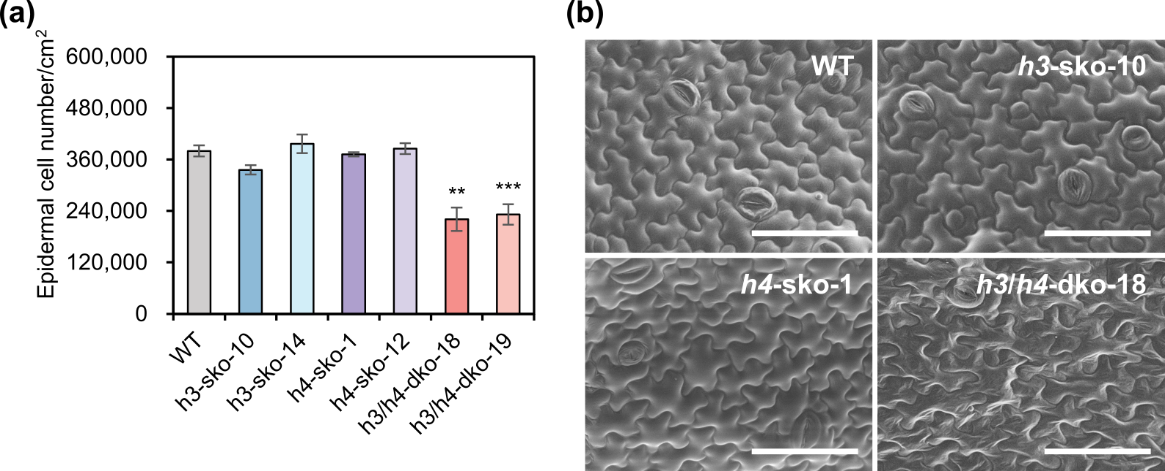


**Fig. S9. Epidermal cells on leaves of *h3* sko, *h4* sko, and *h3*/*h4* dko plants.**

(**a**) Densities of epidermal cells on leaves of WT and ko plants. Data are presented as the mean (±SE) of six biological replicates. ***P* < 0.01, ****P* < 0.001 (unpaired Student’s *t*-test). (**b**) SEM micrographs of the leaf epidermis. Scale bar: 50 μm.

**
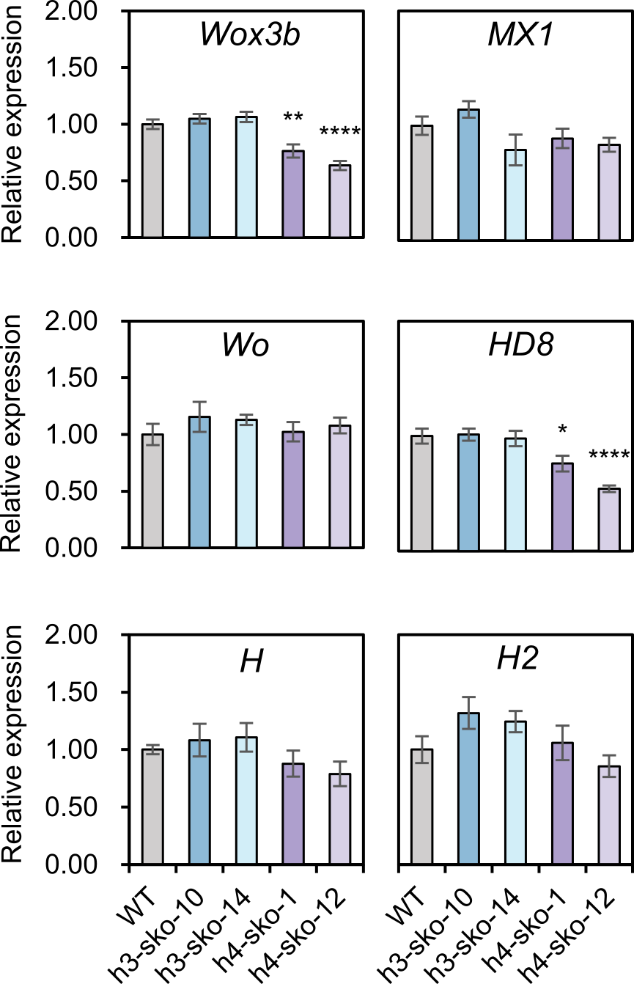
**

**Fig. S10.** **qRT-PCR analysis of trichome-related genes in leaves of *h3* sko and *h4* sko plants.**

Expression values were normalized to those of WT plants. Data are presented as the mean (±SE) of three biological replicates, each comprising three pooled leaf samples. **P* < 0.05, ***P* < 0.01, ***P* < 0.01, *****P* < 0.0001 (unpaired Student’s *t*-test).

**
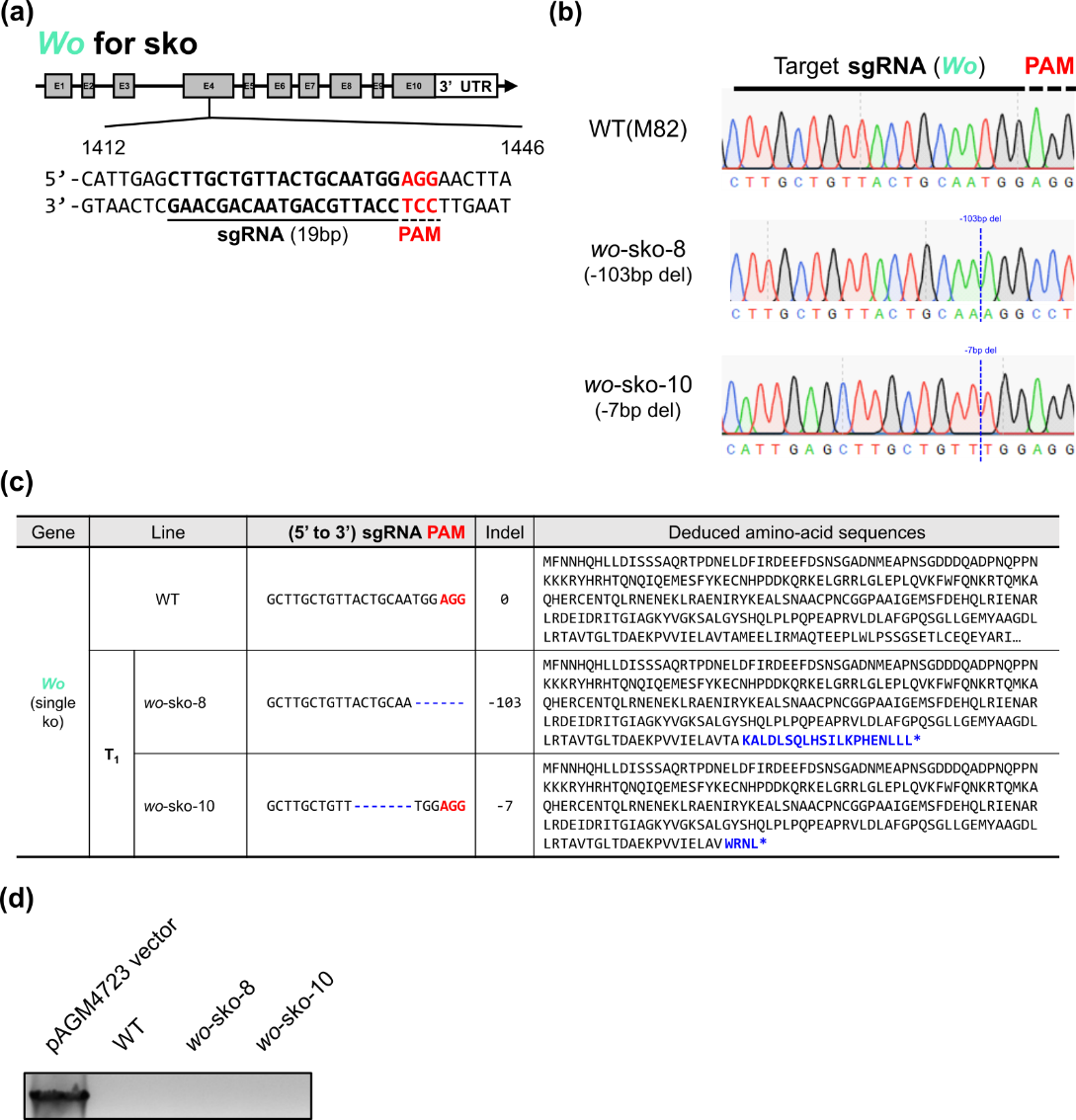
**

**Fig. S11. Development of *wo* sko plants using the CRISPR-Cas9 system.**

(**a**) Gene structures and sgRNA target sites of *Wo* for generating *wo* sko plants. (**b**) Sequence analysis of *wo* sko T_1_ plants. The modified nucleotides in *wo* sko plants are highlighted with a blue cursor (deletion). (**c**) Genotypes and deduced amino acid sequences of *wo* sko T_1_ plants. The nucleotides colored in black and red represent the sgRNA and PAM sequences, respectively. Blue letters and hyphens in the nucleotide sequences of ko plants indicate nucleotide insertions and deletions, respectively. Blue letters in the amino acid sequences of ko plants indicate modified amino acid sequences compared to those of WT plants. Asterisks denote a premature stop codon. (**d**) Selection of transgene-free *wo* sko T_1_ lines. The agarose gel image indicates the presence or absence of the PCR-amplified *Cas9* gene from the gDNA of *wo* sko plants. The pAGM4723 vector served as a positive control, while the gDNA of WT plants was used as a negative control.

**
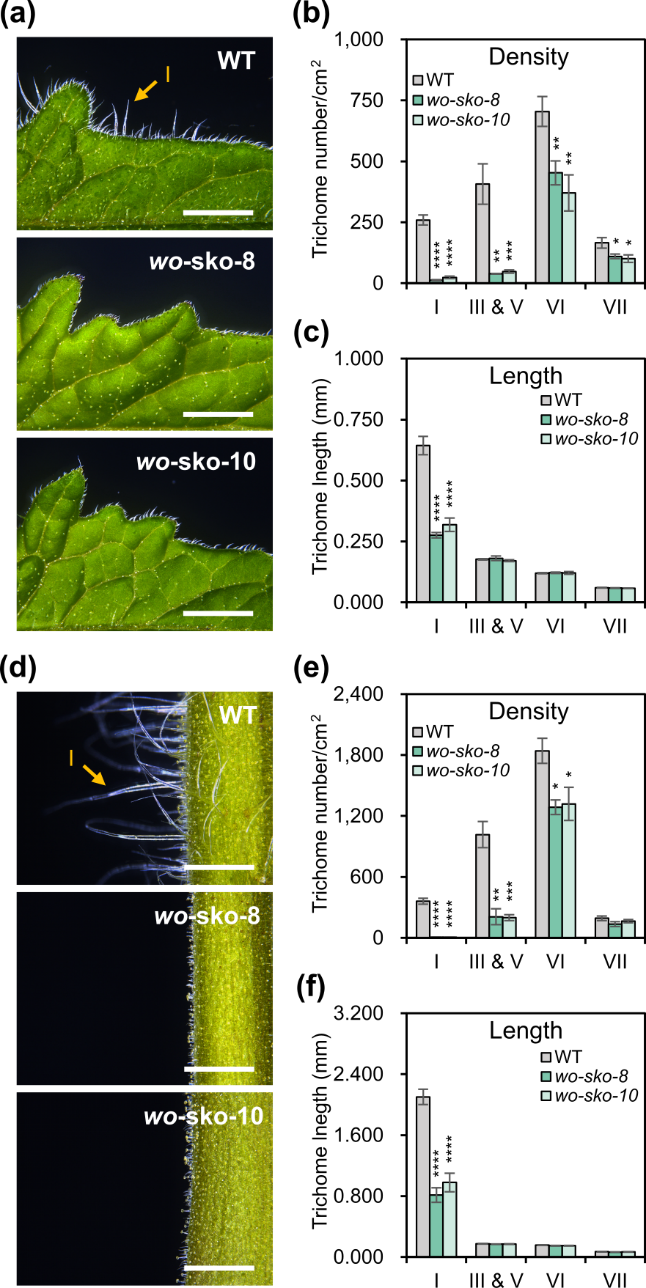
**

**Fig. S12. Trichome phenotypes of *wo* sko plants.**

(**a**) Dissection microscopy (DM) images of leaves in WT and *wo* sko plants. Scale bars: 2 mm. (**b, c**) Trichome density (b) and length (c) on leaves in WT and *wo* sko plants. (**d**) DM images of stems in WT and *wo* sko plants. Scale bars: 2 mm. (**e, f**) Trichome density (e) and length (f) on stems in WT and *wo* sko plants. All images were taken from six-week-old plants. Data are presented as the mean (±SE) of six biological replicates. Asterisks indicate significant differences between WT and *wo* sko plants (unpaired Student’s *t*-test: **P* < 0.05; ***P* < 0.01; ****P* < 0.001; *****P* < 0.0001).


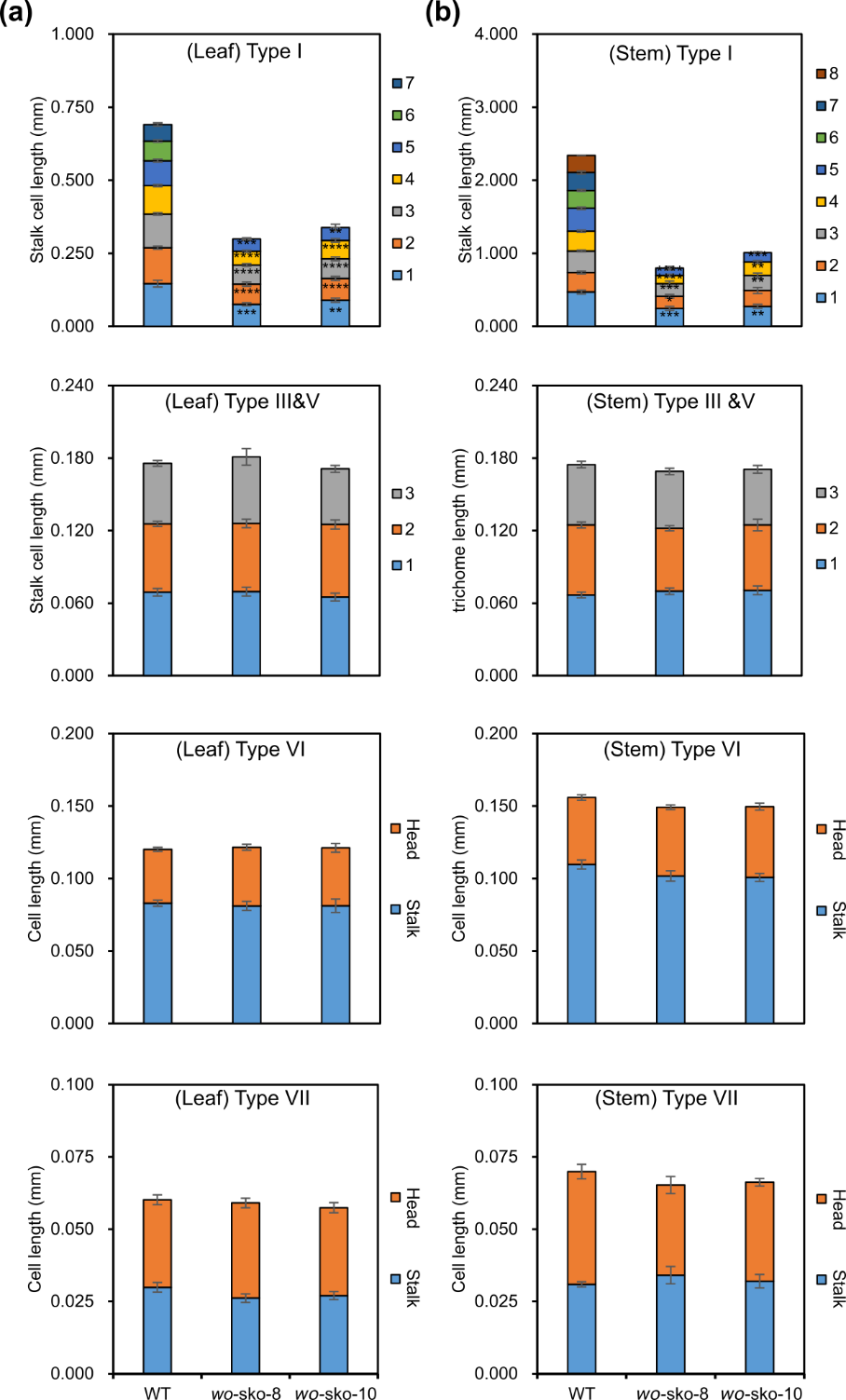


**Fig. S13. Analysis of trichome cell number and length in *wo* sko plants.**

(**a, b**) Cell number and length of each cell type within trichomes on leaves (a) and stems (b) in WT and *wo* sko plants. Numbers on the right indicate stalk cell order from bottom (1) to top (7 or 8 for type I trichomes; 3 for type III and V trichomes). “Stalk” and “Head” refer to the stalk and glandular head cells of type VI and VII trichomes, respectively. Data are presented as the mean (±SE) of three biological replicates. Asterisks indicate significant differences between WT and *wo* sko plants (unpaired Student’s *t*-test: **P* < 0.05; ***P* < 0.01; ****P* < 0.001; *****P* < 0.0001).


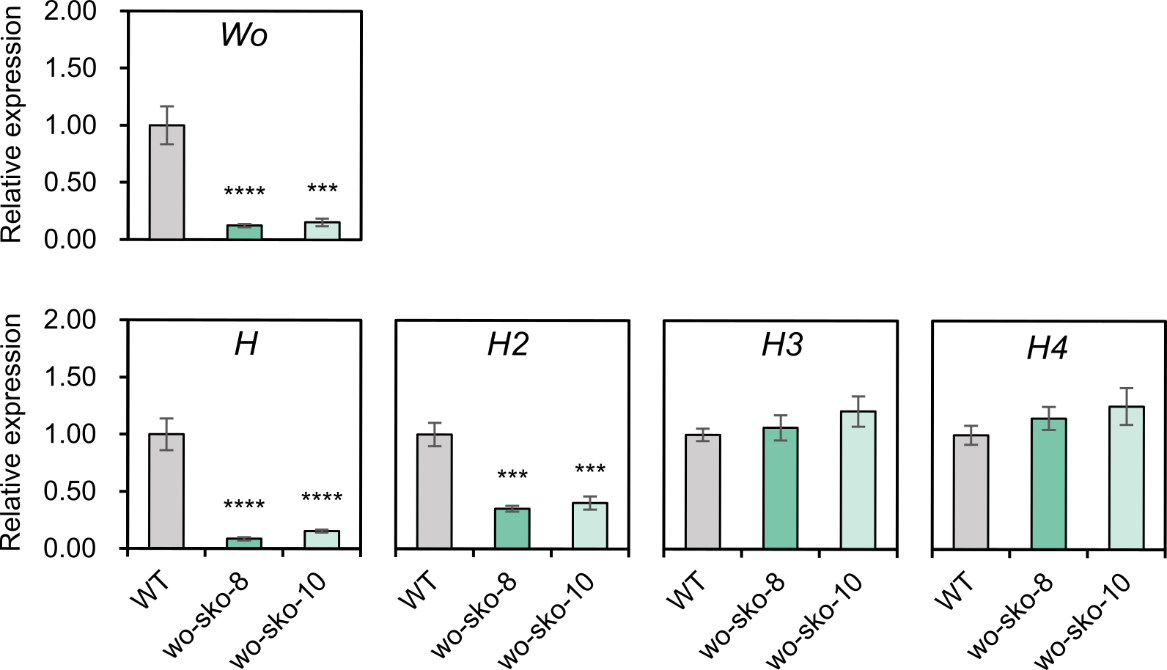


**Fig. S14. qRT-PCR analysis of *Wo* and *ZFPs* in the leaves of *wo* sko plants.**

Expression values were normalized to those of WT plants. Data are presented as the mean (±SE) of three biological replicates, each comprising three pooled leaf samples. ****P* < 0.001, *****P* < 0.0001 (unpaired Student’s *t*-test).


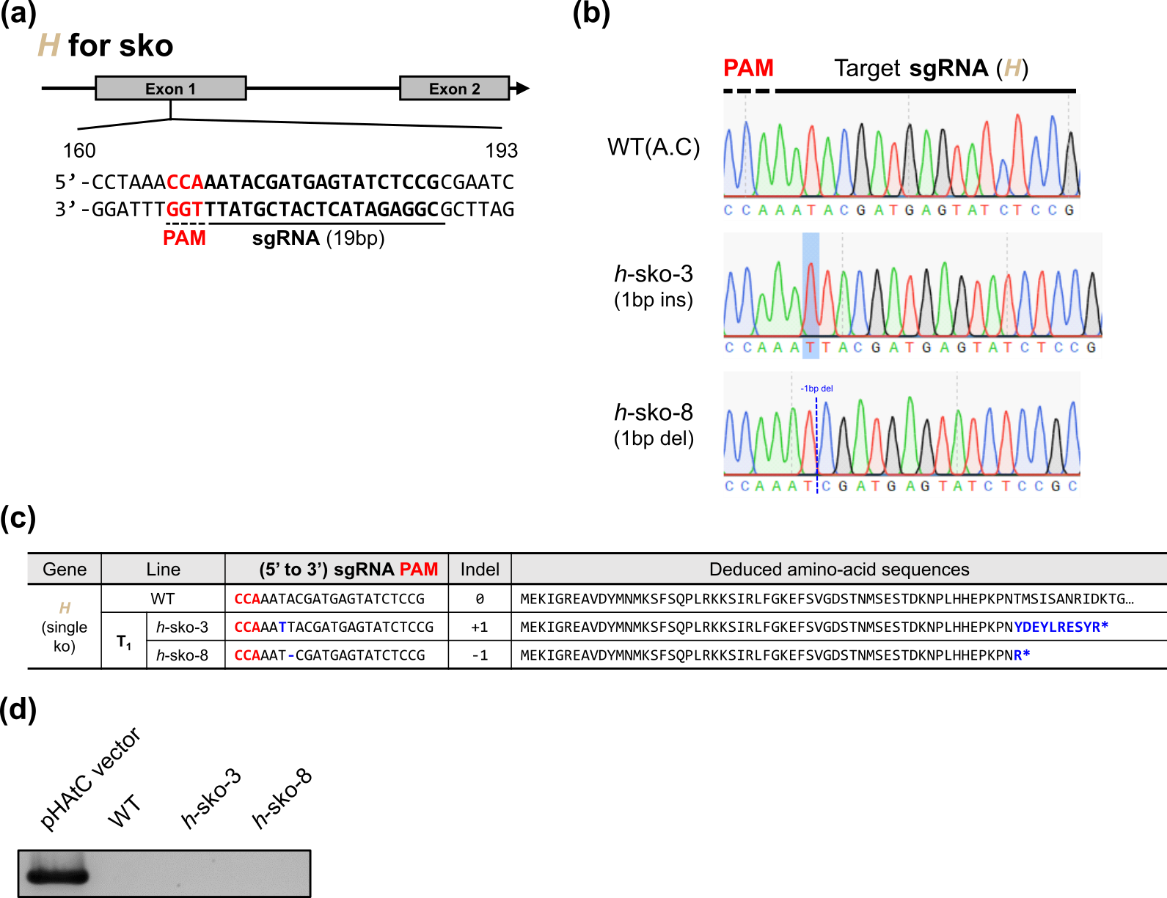


**Fig. S15. Development of *h* sko plants using the CRISPR-Cas9 system.**

(**a**) Gene structures and sgRNA target sites of *H* for generating *h* sko plants. (**b**) Sequence analysis of *h* sko T_1_ plants. The modified nucleotides in *h* sko plants are highlighted with a blue background (insertion) or cursor (deletion). (**c**) Genotypes and deduced amino acid sequences of *h* sko T_1_ plants. The nucleotides colored in black and red represent the sgRNA and PAM sequences, respectively. Blue letters and hyphens in the nucleotide sequences of ko plants indicate nucleotide insertions and deletions, respectively. Blue letters in the amino acid sequences of ko plants indicate modified amino acid sequences compared to those of WT plants. Asterisks denote a premature stop codon. (**d**) Selection of transgene-free *h* sko T_1_ lines. The agarose gel image indicates the presence or absence of the PCR-amplified *Cas9* gene from the gDNA of *wo* sko plants. The pHAtC vector served as a positive control, while the gDNA of WT plants was used as a negative control.


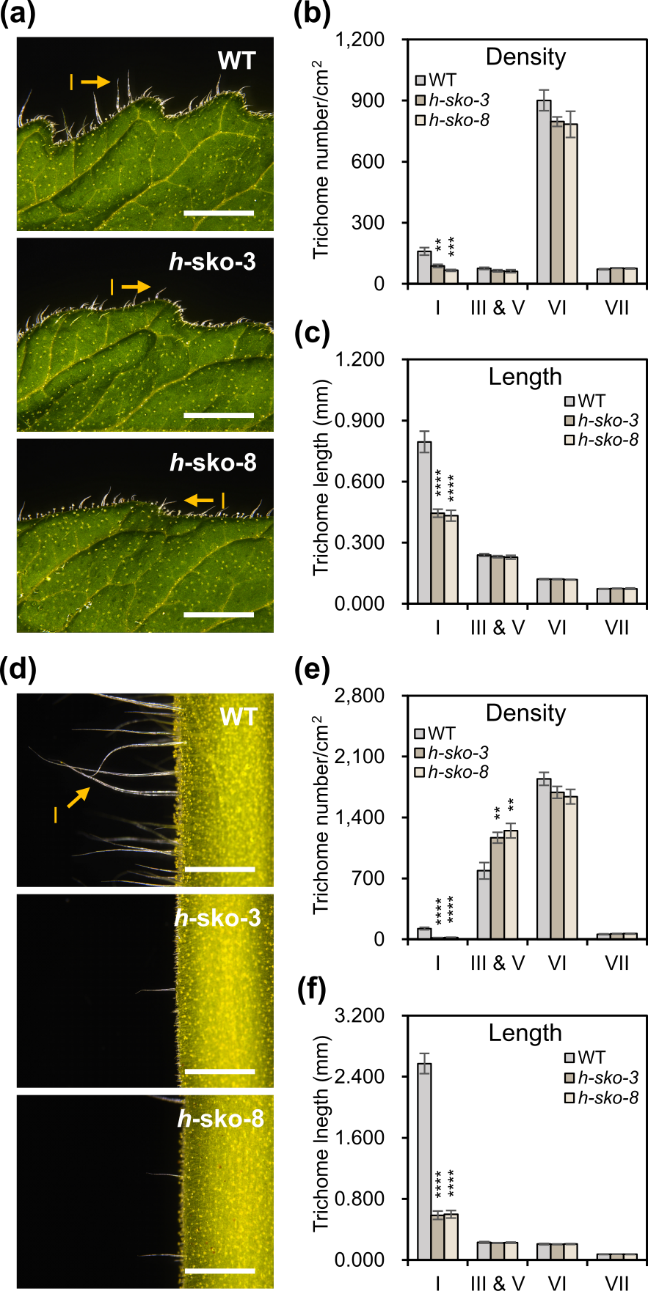


**Fig. S16. Trichome phenotypes of *h* sko plants.**

(**a**) Dissection microscopy (DM) images of leaves in WT and *h* sko plants. Scale bars: 2 mm. (**b, c**) Trichome density (b) and length (c) on leaves in WT and *h* sko plants. (**d**) DM images of stems in WT and *h* sko plants. Scale bars: 2 mm. (**e, f**) Trichome density (e) and length (f) on stems in WT and *h* sko plants. All images were taken from 6-week-old plants. Data are presented as the mean (±SE) of six biological replicates. Asterisks indicate significant differences between WT and *h* sko plants (unpaired Student’s *t*-test: ***P* < 0.01; ****P* < 0.001; *****P* < 0.0001).


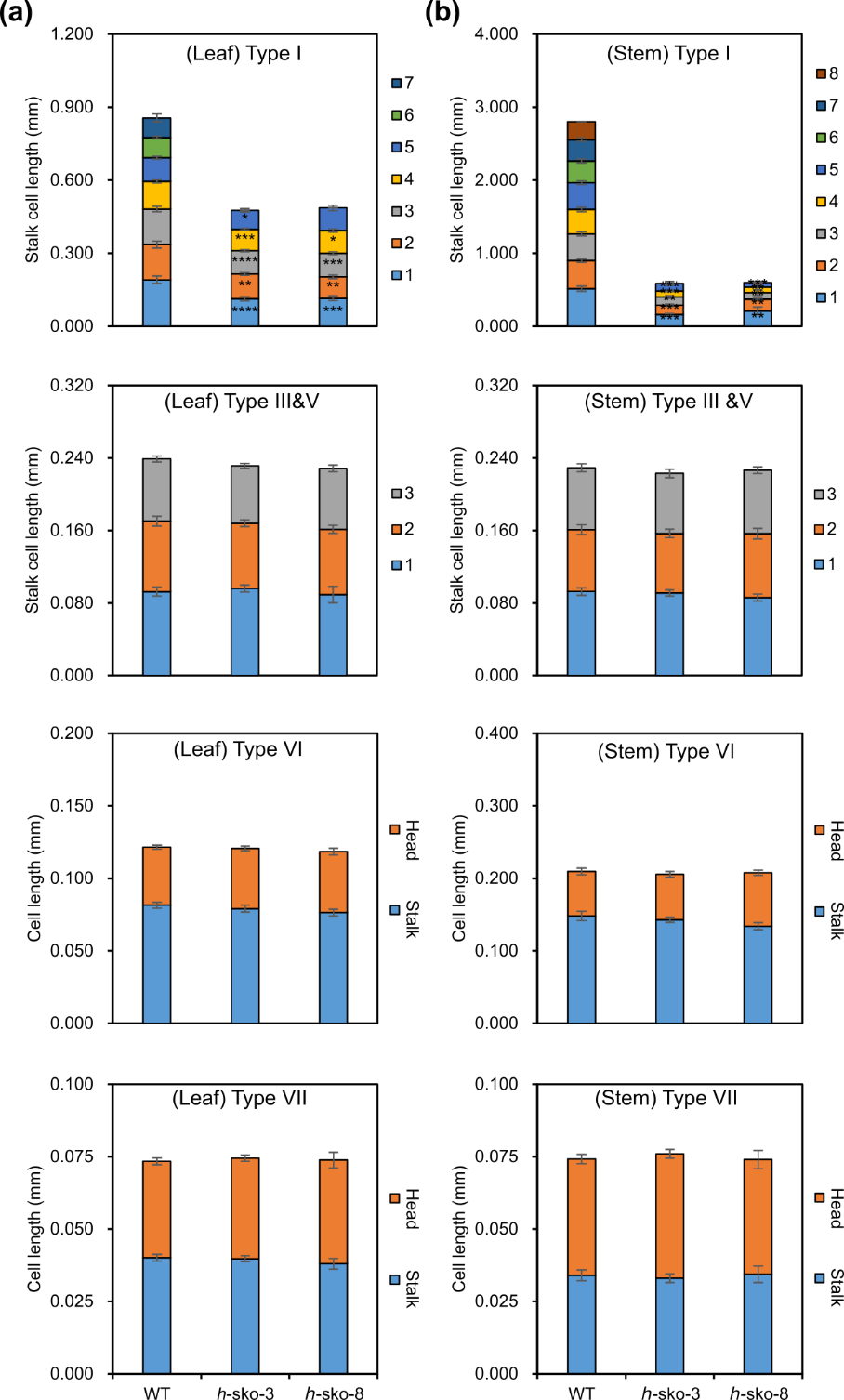


**Fig. S17. Analysis of trichome cell number and length in *h* sko plants.**

(**a, b**) Cell number and length of each cell type within trichomes on leaves (a) and stems (b) in WT and *h* sko plants. Numbers on the right indicate stalk cell order from bottom (1) to top (7 or 8 for type I trichomes; 3 for type III and V trichomes). “Stalk” and “Head” refer to the stalk and glandular head cells of type VI and VII trichomes, respectively. Data are presented as the mean (±SE) of three biological replicates. Asterisks indicate significant differences between WT and *wo* sko plants (unpaired Student’s *t*-test: **P* < 0.05; ***P* < 0.01; ****P* < 0.001; *****P* < 0.0001).


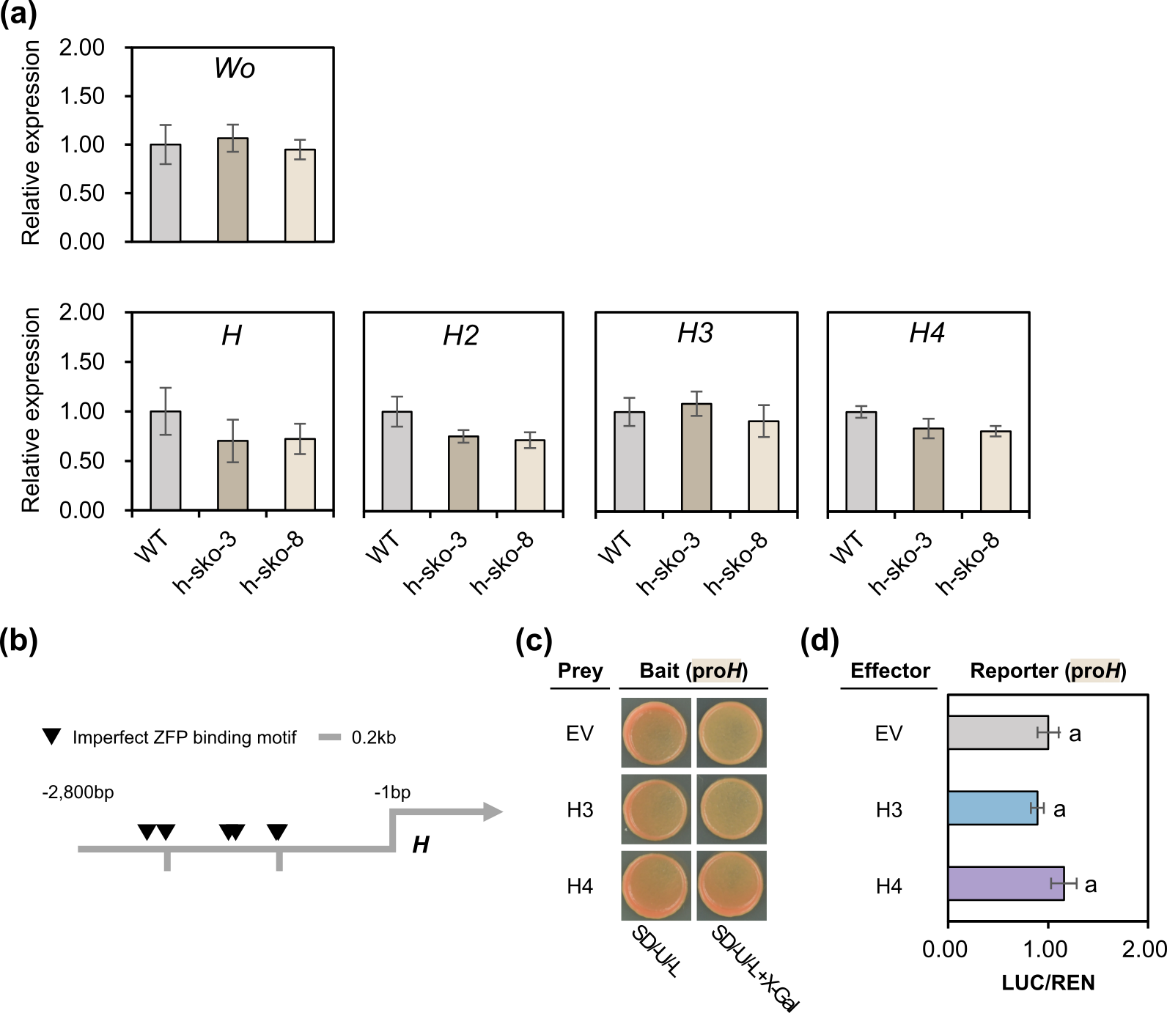


**Fig. S18. H3 and H4 indirectly regulate *H* expression.**

(**a**) qRT-PCR analysis of *Wo* and *SlZFPs* in the leaves of *h* sko plants. Expression values were normalized to those of WT plants. Data are presented as the mean (±SE) of three biological replicates, each comprising three pooled leaf samples. (**b**) Schematic representation of the promoter region (2.8 kb) of *H*. Black triangles indicate imperfect ZFP binding sites. Scale bar: 200 bp. (**c**) Analysis of H3 and H4 binding to the *H* promoter using Y1H. The *H* promoter was fused to the pLacZi bait vector (pLacZi-pro*H*). Full-length H3 and H4 were fused downstream of the GAL4 DNA AD in pGADT7. Constructs containing the pGADT7-EV were used as negative controls. Linearized bait vectors were integrated into the yeast genome, and prey vectors were transformed into the corresponding yeast strain, YM4271. The transformants were incubated on an SD/-Ura/-Leu dropout medium for 3 d and transferred onto an SD/-Ura/-Leu dropout medium containing X-gal. (**d**) Analysis of *H* promoter transactivation by H3 and H4 proteins using DLR assay. The *H* promoter was cloned upstream of LUC into the pGreen II-0800-LUC reporter vector (pGreen II-pro*H*). Full-length H3 and H4 were fused downstream of the 35S promoter in the pKCo effector vectors (*H3* and *H4*, respectively). Both reporter and effector constructs were co-infiltrated in tobacco leaves via agroinfiltration. Constructs containing an empty pKCo vector (35S:EV) were used as negative controls. The DLR assay was performed 2 d post-inoculation. Luciferase activity was quantified using the value of LUC relative to that of each reporter vector and normalized to that of the negative control. Data are presented as the mean (±SE) of eight biological replicates. Different letters indicate statistically significant differences (*P* < 0.05, one-way ANOVA with Tukey’s *post hoc* test).

**
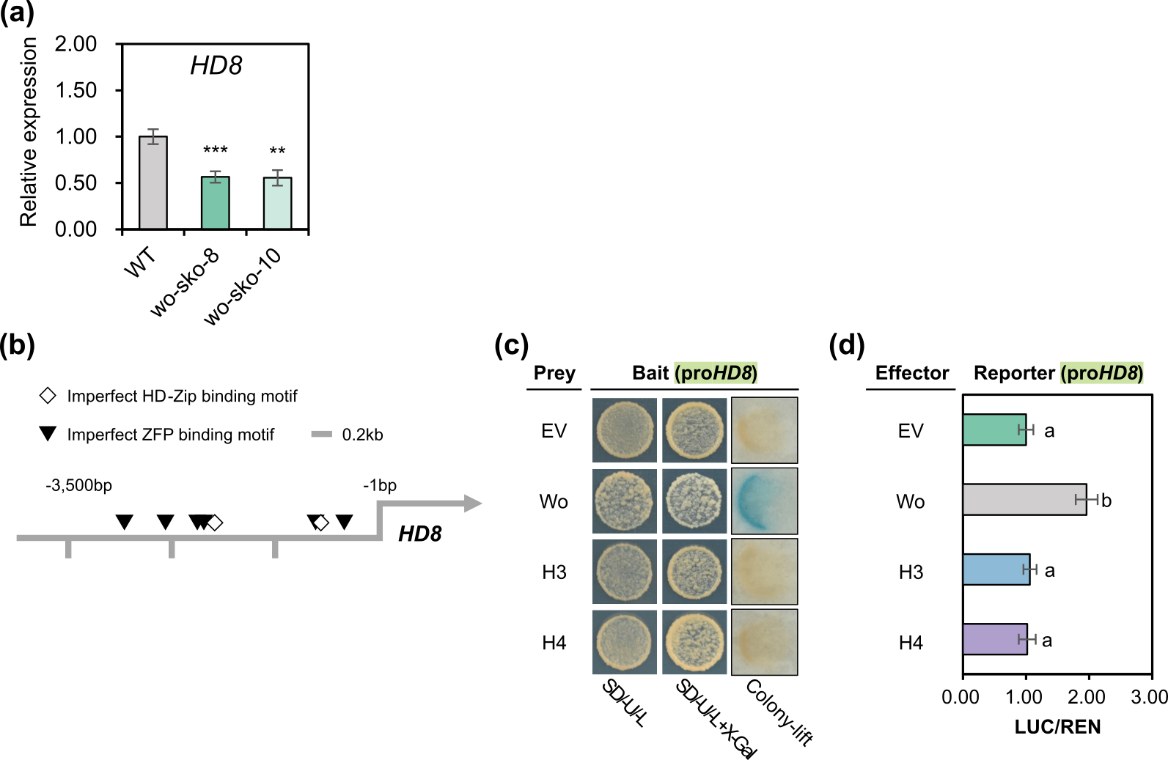
**

**Fig. S19. Wo directly regulates *HD8* expression.**

(**a**) qRT-PCR analysis of *HD8* in the leaves of *wo* sko plants. Expression values were normalized to those of WT plants. Data are presented as the mean (±SE) of three biological replicates, each comprising three pooled leaf samples. (**b**) Schematic representation of the promoter region (3.3 kb) of *HD8*. White diamond and black triangles indicate imperfect HD-Zip (L1 box) and ZFP binding sites, respectively. Scale bar: 200 bp. (**c**) Analysis of Wo, H3, and H4 binding to the *HD8* promoter using Y1H. The *HD8* promoter was fused to the pLacZi bait vector (pLacZi-pro*HD8*). Full-length Wo, H3, and H4 were fused downstream of the GAL4 DNA AD in pGADT7. Constructs containing the pGADT7-EV were used as negative controls. Linearized bait vectors were integrated into the yeast genome, and prey vectors were transformed into the corresponding yeast strain, YM4271. Colonies grown on SD/-Ura/-Leu for 3d were transferred to SD/-Ura/-Leu with X-gal for in vivo and colony-lift filter assays. (**d**) Analysis of *HD8* promoter transactivation by Wo, H3, and H4 proteins using DLR assay. The *HD8* promoter was cloned upstream of LUC into the pGreen II-0800-LUC reporter vector (pGreen II-pro*HD8*). Full-length Wo, H3, and H4 were fused downstream of the 35S promoter in the pKCo effector vectors (*H3* and *H4*, respectively). Both reporter and effector constructs were co-infiltrated in tobacco leaves via agroinfiltration. Constructs containing an empty pKCo vector (35S:EV) were used as negative controls. The DLR assay was performed 2 d post-inoculation. Luciferase activity was quantified using the value of LUC relative to that of each reporter vector and normalized to that of the negative control. Data are presented as the mean (±SE) of eight biological replicates. Different letters indicate statistically significant differences (*P* < 0.05, one-way ANOVA with Tukey’s *post hoc* test).

**
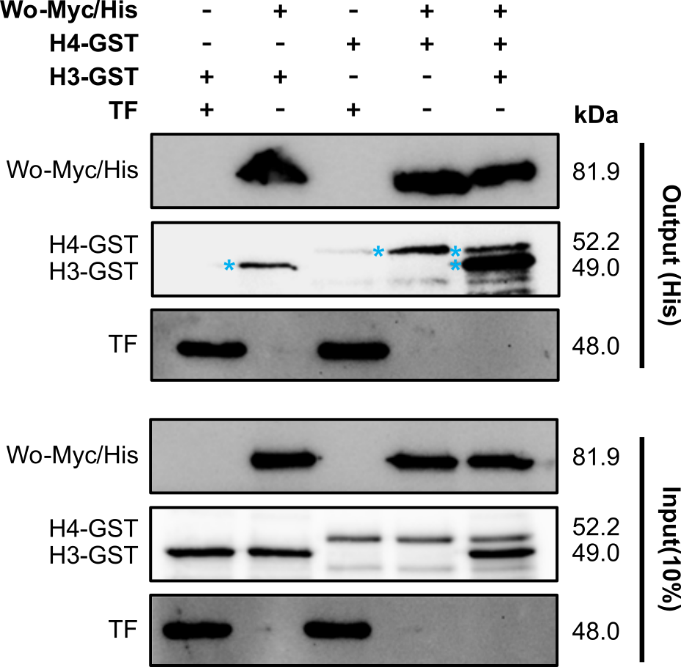
**

**Fig. S20. Protein–protein interactions among Wo, H3, and H4.**

*In vitro* pull-down assays were performed by incubating Wo-Myc/His protein with H3-GST, H4-GST, or both proteins. Proteins bound to Ni-NTA agarose were analyzed via immunoblots using anti-GST, anti-Myc, and anti-TF antibodies. TF served as a negative control. Blue asterisks denote the expected sizes of H3-GST or H4-GST.
